# Efficacy and biomarker analysis of nivolumab plus gemcitabine and cisplatin in patients with unresectable or metastatic biliary tract cancers: results from a phase II study

**DOI:** 10.1136/jitc-2019-000367

**Published:** 2020-06-02

**Authors:** Kaichao Feng, Yang Liu, Yongtian Zhao, Qingming Yang, Liang Dong, Jiejie Liu, Xiang Li, Zhikun Zhao, Qian Mei, Weidong Han

**Affiliations:** 1 Department of Bio-therapeutic, the First Medical Center, Chinese PLA General Hospital, Beijing, China; 2 Department of Geriatric Hematology, the Second Medical Center, Chinese PLA General Hospital, Beijing, China; 3 YuceBio Technology Co., Ltd, Shenzhen, China

**Keywords:** immunology, oncology

## Abstract

**Background:**

The prognosis of patients with unresectable or metastatic biliary tract cancer (BTC) is unacceptably low. This study aimed to determine the efficacy, safety and predictive biomarkers of the immune checkpoint inhibitor nivolumab in combination with chemotherapy in advanced BTCs.

**Methods:**

In this open-label, single-arm, phase II trial, a chemotherapy and immunotherapy combination consisting of gemcitabine 1000 mg/m^2^, cisplatin 75 mg/m^2^ and nivolumab 3 mg/kg was administered every 3 weeks for up to six cycles. Maintenance treatment with gemcitabine plus nivolumab was administered to patients achieving disease control following the combination therapy. The primary outcome was the objective response rate. Secondary outcomes included safety, disease control rate (DCR), progression-free survival (PFS) and overall survival (OS). The exploratory objective was to assess biomarkers for predicting clinical response and prognosis.

**Results:**

Thirty-two patients with a median age of 60 (range 27–69) years were enrolled. As of September 31, 2019, the median follow-up was 12.8 (95% CI 10.8 to 14.8) months. Twenty-seven response-evaluable patients received a median of 4 (IQR, 3–6) cycles of combination therapy, of whom 15 (55.6%) patients achieved an objective response, including 5 (18.6%) with a complete response (CR), and the DCR was 92.6%. Of the six patients in cohort A who were resistant to gemcitabine-based or cisplatin-based chemotherapy, one achieved CR and one achieved partial response. Thirteen of 21 chemotherapy-naive patients (61.9%) in cohort B achieved an objective response. The median PFS of all patients in cohorts A+B was 6.1 months. The median OS was 8.5 months, with a 33.3% 12-month OS rate. The most frequent grade 3 or higher adverse events were thrombocytopenia (56%) and neutropenia (22%). Fitness might be a biomarker for predicting clinical response. On-therapy changes in serum soluble FasL, MCP-1 and interferon-γ were correlated with prognosis.

**Conclusions:**

Nivolumab in combination with gemcitabine and cisplatin offers promising efficacy and a manageable safety profile for patients with advanced BTCs.

**Trial registration number:**

NCT03311789

## Background

Biliary tract cancers (BTCs) represent a diverse group of highly invasive heterogeneous epithelial cancers arising from the biliary tract with poor prognosis. Based on their anatomical location, BTCs are classified into gallbladder carcinoma, intrahepatic cholangiocarcinoma, perihilar cholangiocarcinoma and distal cholangiocarcinoma. The incidence of BTCs has increased globally over the past few decades,^1^ with 235,900 patients reported to have been diagnosed with BTCs in 2017.^2^ Surgical resection is a curative treatment option for early-stage BTCs; however, most patients with BTCs already have locally advanced or metastatic disease at the time of diagnosis. Even with surgical resection, recurrence is seen in >60% of patients within the first or the second year.^3^ For patients with advanced unresectable or metastatic BTCs, gemcitabine plus cisplatin is the current standard first-line systemic therapy.^4^ However, this combination regimen confers limited efficacy. One possible reason is the rich desmoplastic stroma of BTCs, which forms a barrier to the delivery of chemotherapeutic drugs in the tumor bed and results in resistance to chemotherapy. Other regimens or strategies, such as gemcitabine and oxaliplatin with or without cetuximab,^5^ capecitabine plus cisplatin,^6^ nab-paclitaxel and gemcitabine,^7^ and small-molecule kinase inhibitors targeting FGFR, IDH, MET, mesothelin, BRCA and some mutated proteins, did not show significant improvements in efficacy and survival.^8 9^

Recently, immune checkpoint inhibitors (ICIs), exemplified by antibodies targeting programmed cell death-1 (PD-1) and programmed cell death-ligand 1 (PD-L1), have demonstrated promising antitumor activity in a variety of tumor types, coupled with low rates of immune-mediated toxicity.^10 11^ However, studies of anti-PD-1/PD-L1 antibodies in BTCs are limited. The KEYNOTE-028 trial reported that 17% of patients with PD-L1-positive advanced BTCs obtained partial response (PR) from pembrolizumab monotherapy.^12^ In another basket trial, pembrolizumab resulted in 100% disease control in four patients with tumor DNA mismatch repair (MMR)-deficient cholangiocarcinoma.^13^ However, MMR deficiency occurred in only 5%–10% of patients with BTCs.^14^ Therefore, novel strategies that could improve the efficacy of ICIs are urgently needed.

Many studies have demonstrated that ICIs can interact synergistically with chemotherapy in solid tumors.^15^ However, there have been few reports of this combination therapy in advanced BTCs. Here, we conducted a phase II trial to evaluate the efficacy, safety and biomarkers of nivolumab in combination with gemcitabine and cisplatin for advanced unresectable or metastatic BTCs.

## Methods

### Study design and patients

This study was a single-center, single-arm, open-label, phase Ⅱ trial in which the key inclusion criteria were aged 18–75 years, histologically confirmed unresectable or metastatic BTC, an Eastern Cooperative Oncology Group performance status of 0–2, an estimated life expectancy of at least 3 months, at least one radiographically measurable lesion, adequate organ function, and ability to understand and sign a written informed consent document. Previous chemotherapy, radiotherapy, or other local ablative therapies must have been completed over 4 weeks before enrollment and patients must have shown radiologically confirmed disease progression. The key exclusion criteria included active, known or suspected autoimmune disease, known brain metastasis or active central nervous system disease, being treated with either corticosteroids (>10 mg daily prednisone equivalent) or other immunosuppressive medications within 14 days of enrollment, and previous treatment with anti-PD-1/PD-L1 antibodies. Details of the inclusion and exclusion criteria are presented in the study protocol (online supplementary file 1). Eligible patients were assigned to cohort A (resistant to gemcitabine-based or cisplatin-based chemotherapy) and cohort B (chemotherapy-naive) based on their previous systemic therapies.

10.1136/jitc-2019-000367.supp1 Supplementary data

Written informed consent based on Declaration of Helsinki principles was provided by patients or their representatives before study entry.

### Treatment and assessments

All enrolled patients in both cohort A and cohort B were administered the combination therapy consisting of gemcitabine 1000 mg/m^2^ on day 1 and day 5, cisplatin 75 mg/m^2^ on day 1, and nivolumab 3 mg/kg on day 3 infused intravenously every 3 weeks for up to six cycles. Afterwards, patients with responsive or stable disease (SD) switched to maintenance therapy in which nivolumab and gemcitabine were administered every 6–8 weeks until disease progression, intolerable toxicity, death, withdrawal of consent, or any other reasons. Dose reductions were permitted according to the protocol. Adverse events were graded in accordance with National Cancer Institute Common Terminology Criteria for Adverse Events version 5.0, and the causal association with study drugs was determined by investigators. Tumor responses were assessed every two cycles by site investigators according to Response Evaluation Criteria in Solid Tumors (RECIST) version 1.1.^16^ Positron emission tomography-CT was mandated to confirm response evaluation if targeted tumors were assessed by CT scans with contrast or MRI as showing a complete response (CR). Patients achieving PR or progressive disease (PD) were advised to undergo on-therapy site-matched tumor biopsy. Tumor cell PD-L1 expression was assessed on either archival or fresh pretreatment biopsy samples by immunohistochemistry using the Dako 22C3 pharmDx assay (Dako North America, Carpinteria, California, USA). Positive tumor PD-L1 expression was defined as at least 1% of tumor cells being membrane stained at any intensity in a section that contained at least 100 evaluable tumor cells.

### Whole-exome sequencing

Genomic DNA was isolated from tumor biopsies and matched peripheral blood mononuclear cell samples using the GeneRead DNA FFPE Kit. All sample capture libraries were prepared using the Agilent SureSelect Human All Exon V6 Kit (Agilent Technologies, Santa Clara, California, USA) according to the manufacturer’s instructions. Libraries were sequenced on an Illumina HiSeq 6000 platform. Primary sequence data were processed by filtering adaptor sequences and removing low-quality reads, which were defined as those with a >10% N rate and/or with >10% bases with a quality score of <20 using SOAPnuke (V.1.5.6). The clean reads were mapped to hg19 using BWA-mem (V.0.7.12). Single nucleotide variants and small insertions and deletions (indels) were detected using VarScan (V.2.4.1). The mutations were further filtered using inhouse software to remove false positive mutations. Tumor mutational burden (TMB) was determined by analyzing non-silent somatic mutations, including coding base substitution and indels per megabase. PyClone was employed to detect subclones and calculate the cancer cell fraction. The ratio of these subclones to all mutations was interpreted as intratumor heterogeneity. Microsatellite instability (MSI) detection was performed by interrogating 344 available genomic microsatellites using an MSI sensor. The percentage of unstable sites was reported as the MSI sensor score. Human lymphocyte antigen-I (HLA-I) typing of tumors and adjacent normal samples was performed using Polysolver (V.1.0). All non-silent mutations were translated into 21-mer peptide sequences. Then, 9-mer to 11-mer peptide sequences were extracted using a sliding window. NetMHCpan (V.3.0) was used to predict the major histocompatibility complex (MHC) class I binding affinity of peptides with the patient-specific HLA alleles. The predicted peptides were selected and ranked by inhouse software. Peptides with scores higher than 0 were selected. Tumor neoantigen burden (TNB) was measured as the number of such peptides per megabase. In the neoantigen fitness model, we calculated the neoantigen recognition potential for each neoantigen using a recently developed method.^17^

### Cytokines

Peripheral blood samples were collected every cycle prior to the infusion of study drugs to test the concentration level of cytokines, including interleukin (IL)-1β, IL-2, IL-4, IL-6, IL-8 (CXCL8), IL-10, IL-12p70, IL-17A, IL-18, IL-23, IL-33, interferon (IFN)-α2, IFN-γ, tumor necrosis factor (TNF)-α, soluble Fas, soluble FasL (sFasL), granzyme A, granzyme B, perforin, granulysin, and monocyte chemotactic protein-1 (MCP-1) C-C motif chemokine ligand 2 (CCL2), using BioLegend LEGENDplex bead-based immunoassays, the LEGENDplex Human Inflammation Panel (Cat: 740118), and the Human CD8/NK Panel (Cat: 740267).

### Endpoints

The primary objective of this study was to assess the objective response rate (ORR) for nivolumab plus gemcitabine and cisplatin combination therapy. The secondary objectives included determining the frequency and severity of adverse events occurring up to 120 days after the last dose of study drugs, disease control rate (DCR), progression-free survival (PFS), PFS at 6 months, overall survival (OS), and OS at 12 months. ORR was defined as the proportion of all treated patients with either a confirmed CR or PR per RECIST version 1.1. PFS was defined as the time from the first dose to the first documented disease progression or to death from any cause. OS was defined as the time from the first dose to death from any cause. The exploratory objective was to assess pathological, immunological or clinical predictive biomarkers for response and prognosis.

### Statistical analysis

The A’Hern single-stage phase II study design was used to determine the sample size of this clinical trial of nivolumab plus gemcitabine and cisplatin chemotherapy. According to previously reported data, the ORR of gemcitabine and cisplatin chemotherapy for patients with advanced BTCs is up to 26%. Based on this, we set the null hypothesis to be that 26% or fewer patients would have an objective response versus the alternative hypothesis that it was 55% or higher. At least 25 patients would need to be enrolled with a two-sided significance level of 0.05% and 90% power. Safety analysis was performed in patients who received at least one dose of nivolumab in combination with gemcitabine and cisplatin, and efficacy analysis was performed in patients who underwent one or more post-treatment scans. The proportions of patients with an objective response and adverse events were summarized by descriptive statistics with Wilson 95% CIs. Response differences among clinical subgroups were assessed with Fisher’s exact test. For PFS, patients without disease progression were censored at the time of last radiological imaging. For OS, patients still surviving were censored at the time of data cut-off. Survival was analyzed using the Kaplan-Meier method and compared using the log-rank test. Immune biomarker changes were detected by paired t-tests between pretreatment and post-treatment, and differences among groups were evaluated by t-test or the Mann-Whitney U test. All statistical analyses were completed using Stata/SE V.15.1.

## Results

### Patient population

**Figure 1:**
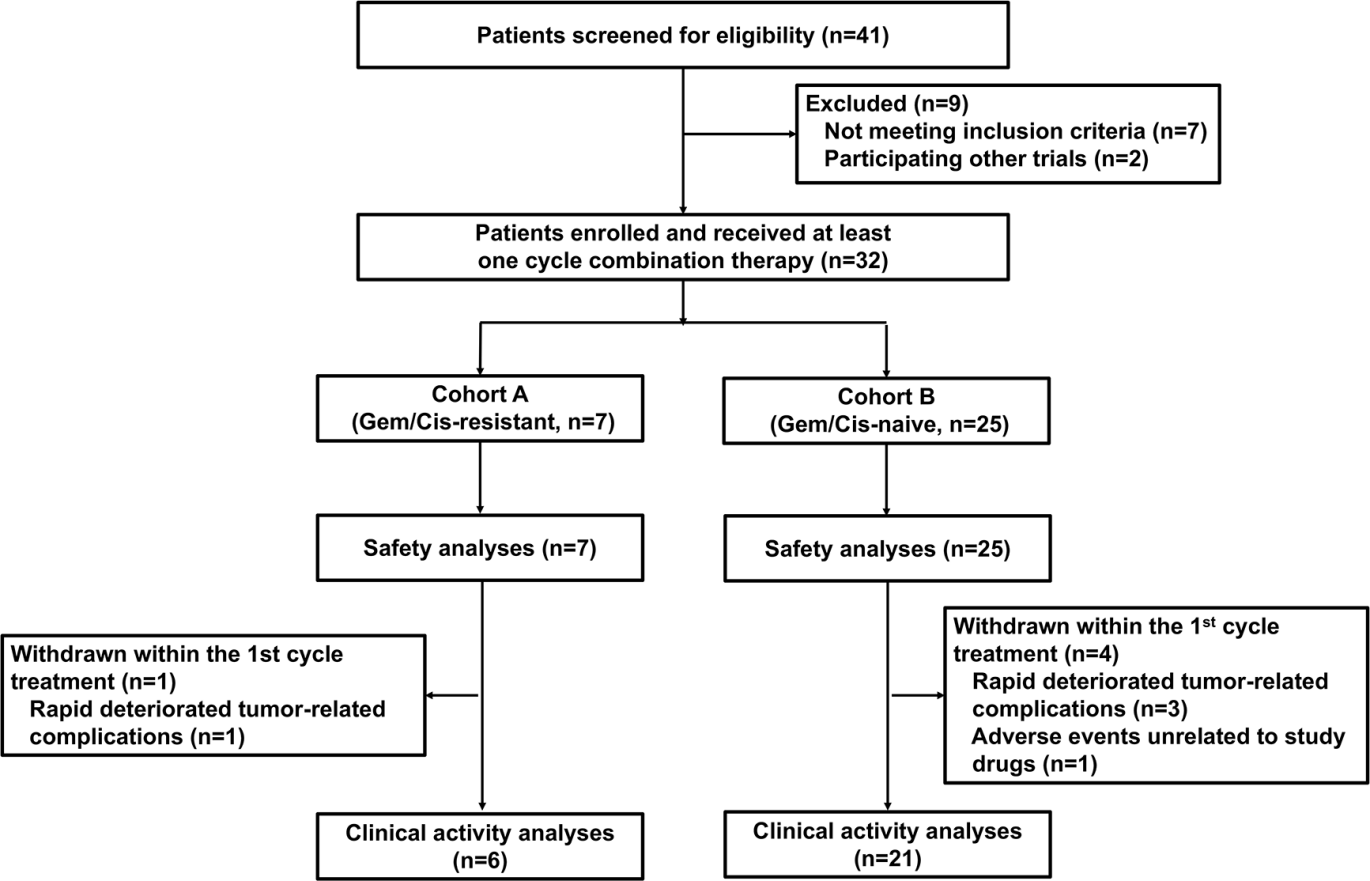
Trial profile. Cis, cisplatin; Gem, gemcitabine.

Between November 16, 2017 and December 31, 2018, 32 eligible patients with advanced unresectable or metastatic BTCs were enrolled, of whom 7 patients were resistant to gemcitabine-based or cisplatin-based chemotherapy and 25 patients were chemotherapy-naive (figure 1). All enrolled patients, including 1 (3%) patient with regional unresectable disease, 6 (19%) with metastatic disease, and 25 (78%) with recurrent disease (defined as patients who had regionally relapsed disease or distant metastases after complete resection or locoregional or systemic therapies), were administered at least one cycle of nivolumab plus gemcitabine and cisplatin combination therapy (table 1). Patients who did not meet the inclusion criteria or were participating in other trials were excluded (n=9). At the time of data cut-off (September 31, 2019), all patients in cohort A and cohort B were eligible for safety analyses, of whom 6 in cohort A and 21 in cohort B were qualified for clinical activity analyses; 5 patients discontinued treatment within the first cycle due to rapidly deteriorating tumor-related complications (1 from cohort A and 3 from cohort B) or adverse events unrelated to study drugs (1 from cohort B). The detailed baseline demographics and characteristics of all enrolled patients are summarized in table 1. The median age was 60 years (range 27–69). Fourteen (44%) patients had target lesions larger than or equal to 5 cm. Liver metastases were detected in 28 (88%) patients, while abdominal lymphatic metastases were detected in 21 (66%). PD-L1 status was evaluable in 26 tumor samples (81%), of which 12 (37%) were positive for PD-L1 expression and 14 (44%) were negative.

**Table 1:** Baseline demographics and characteristics of all enrolled patients

	Patients (N=32)
Median age, years	60 (27–69)
Sex	
Male	18 (56)
Female	14 (44)
Stage at enrollment	
Unresectable	1 (3)
Primary metastatic	6 (19)
Recurrent/metastatic	25 (78)
Histology	
GBCA	6 (19)
Intra-CCA	11 (34)
Perihilar CCA	6 (19)
Distal CCA	9 (28)
ECOG performance status	
0–1	30 (94)
2	2 (6)
Diameter of the largest target lesion (cm)	
<5	18 (56)
≥5	14 (44)
Sum of target lesions (cm)	
<10	20 (62)
≥10	12 (38)
Sites of metastases	
Liver	28 (88)
Lung	5 (16)
Abdominal lymph node	21 (66)
Previous treatment	
Surgery	21 (66)
Locoregional therapy	10 (31)
Chemotherapy	7 (22)
None	7 (22)
Tumor PD-L1 expression	
<1%	14 (44)
≥1%	12 (37)
Not assessable	6 (19)

Data are n (%), unless otherwise specified.

Histology was categorized according to the WHO Classification of Tumors.

CCA, cholangiocarcinoma; ECOG, Eastern Cooperative Oncology Group; GBCA, gallbladder carcinoma; PD-L1, programmed cell death-ligand 1.

### Treatment-related toxicity

Safety data from cohort A and cohort B were summarized and analyzed together. All 32 enrolled patients experienced at least one treatment-related adverse event. The most frequent adverse events were nausea (29 patients; 91%), neutropenia (26 patients; 81%), fatigue (21 patients; 66%), thrombocytopenia (20 patients; 62%), and anemia (19 patients; 59%) (table 2). The most common grade 3 or higher treatment-related adverse events were thrombocytopenia, reported in 18 (56%) patients, and neutropenia, reported in 7 (22%) patients. Other severe adverse events included elevated alanine aminotransferase (1 patient; 3%, grade 3), elevated aspartate aminotransferase (1 patient; 3%, grade 4), elevated lipase (1 patient; 3%, grade 3), hyponatremia (1 patient; 3%, grade 3), and hypertension (2 patients; 6%, grade 3). One (3%) patient had immune-related adverse events (rash, grade 1). There were no treatment-related deaths at the time of analysis.

**Table 2:** Treatment-related adverse events in 32 patients

Treatment-related events	Any grade	Grade 1–2	Grade 3	Grade 4
Anemia	19 (59)	18 (56)	1 (3)	–
Neutropenia	26 (81)	19 (59)	6 (19)	1 (3)
Thrombocytopenia	20 (62)	2 (6)	7 (22)	11 (34)
Nausea	29 (91)	29 (91)	–	–
Vomit	4 (13)	4 (13)	–	–
Constipation	7 (22)	7 (22)		
Fatigue	21 (66)	21 (66)	–	–
Rash	1 (3)	1 (3)	–	–
Fever	11 (34)	11 (34)	–	–
Elevated alanine aminotransferase	9 (28)	8 (25)	1 (3)	–
Elevated aspartate aminotransferase	9 (38)	8 (25)	–	1 (3)
Elevated amylase	1 (3)	1 (3)	–	–
Elevated lipase	2 (6)	1 (3)	1 (3)	–
Hyponatremia	1 (3)	–	–	1 (3)
Peripheral neuropathy	2 (6)	2 (6)	–	–
Hypertension	2 (6)	–	2 (6)	–

Data are n (%), unless otherwise specified.

No patients had fatal treatment-related adverse events.

### Clinical response and biomarkers

**Figure 2:**
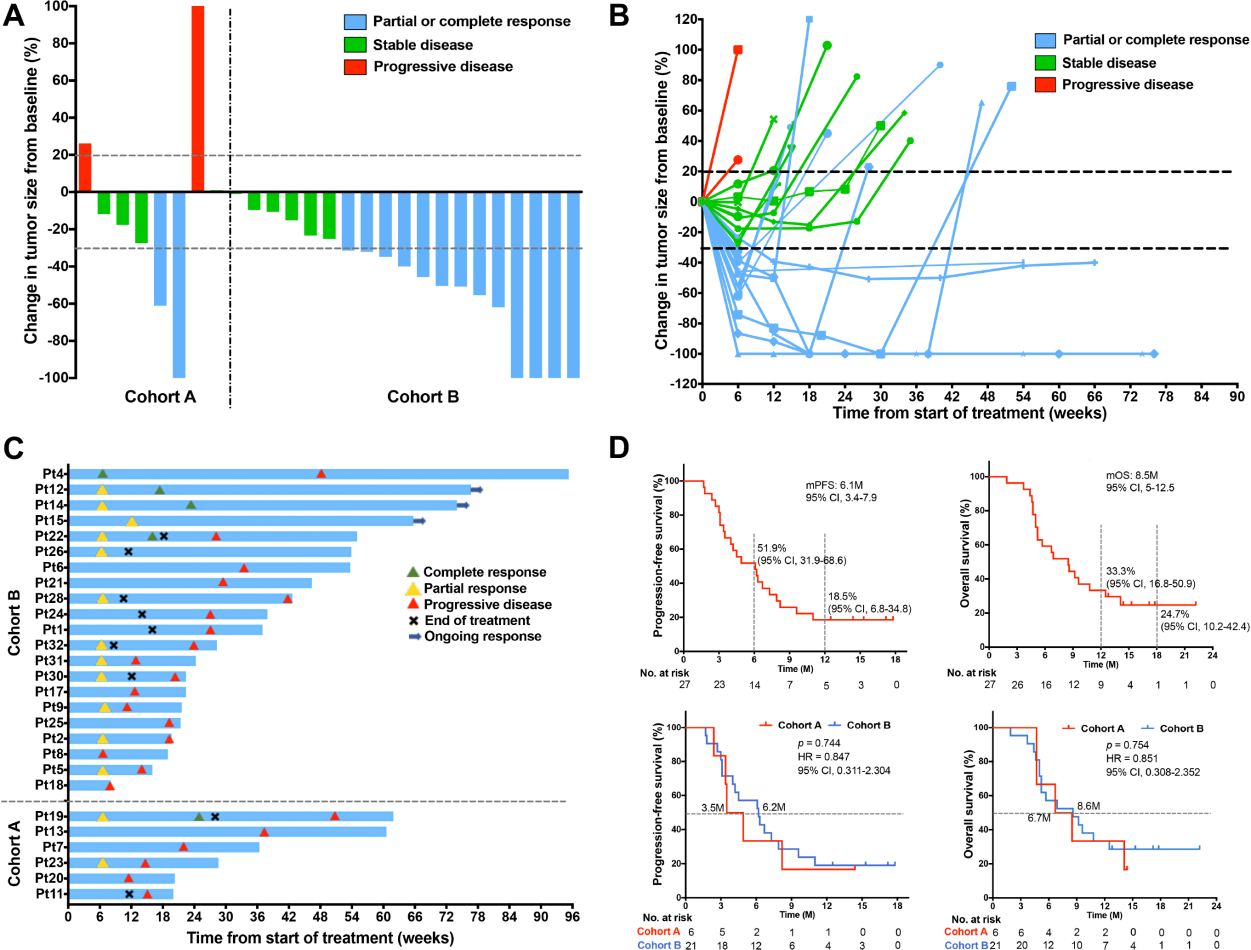
Characteristics of clinical response and survival. (A) Highest percentage change in the change in target lesion size from baseline in patients from cohort A and cohort B; horizontal dotted lines denote a 30% decrease and 20% increase, indicating objective response and progressive disease, respectively, as per RECIST version 1.1. (B) Percentage change in target lesion tumor size from baseline over time for all evaluable patients, defined as those with baseline tumor assessments and at least one post-treatment assessment. The upper horizontal dotted line indicates disease progression at a 20% increase in the size of target lesions, and the lower dotted line represents an objective response at a 30% decrease in the size of target lesions. (C) Time to response and duration of response in patients from cohort A and cohort B. (D) Kaplan-Meier curves of investigator-assessed progression-free survival in all evaluable patients (upper left). Kaplan-Meier curves of investigator-assessed overall survival in all evaluable patients (upper right). Comparison of the median progression-free survival (mPFS) between cohort A and cohort B (low left). Comparison of the median overall survival (mOS) between cohort A and cohort B (low right). RECIST, Response Evaluation Criteria in Solid Tumors.

**Figure 3:**
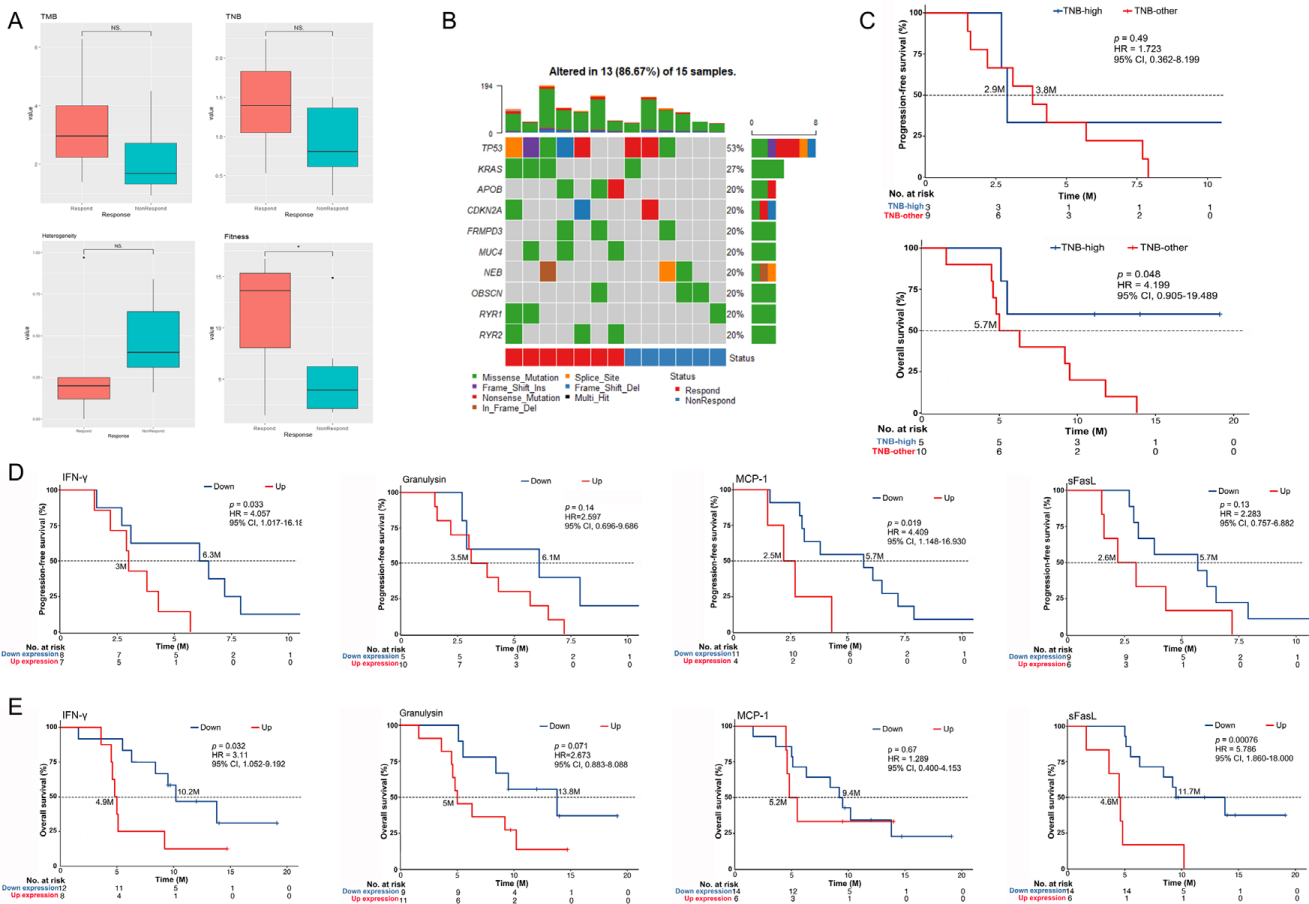
Biomarkers for response and prognosis. (A) Correlation of TMB, TNB, heterogeneity, fitness and clinical response between the responding group and the non-responding group. (B) Mutated genes detected by whole-exome sequencing. (C) Kaplan-Meier curves of progression-free survival and overall survival between patients with high TNB and other levels of TNB. (D) Kaplan-Meier curves of progression-free survival of patients who had increased IFN-γ versus those who had decreased IFN-γ, patients who had increased granulysin versus those who had decreased granulysin, patients who had increased MCP-1 versus those who had decreased MCP-1, and patients who had increased sFasL versus those who had decreased sFasL. (E) Kaplan-Meier curves of overall survival of patients who had increased IFN-γ versus those who had decreased IFN-γ, patients who had increased granulysin versus those who had decreased granulysin, patients who had increased MCP-1 versus those who had decreased MCP-1, and patients who had increased sFasL versus those who had decreased sFasL. IFN-γ, interferon-γ; sFasL, soluble FasL; TMB, tumor mutational burden; TNB, tumor neoantigen burden.

After a median follow-up of 12.8 months (95% CI 10.8 to 14.8), 27 response-evaluable patients in cohort A and cohort B received a median of 4 cycles of nivolumab plus gemcitabine and cisplatin combination therapy (IQR, 3–6). Fifteen (55.6%) patients achieved a confirmed objective response, including 5 (18.6%) CRs and 10 (37%) PRs (table 3, figure 2A). Disease control was achieved in 25 (92.6%) patients, including 10 (37%) patients who had SD as their best response. The radiological changes of each response-evaluable patient are summarized in online supplementary file 2. In cohort A, six of seven patients who were resistant to gemcitabine-based or cisplatin-based regimens were response-evaluable, of whom one patient achieved CR and one patient obtained PR; the ORR and DCR were 33.3% and 83.3%, respectively. In cohort B, 13 of 21 (61.9%) chemotherapy-naive patients achieved CR or PR, and the proportion of patients with disease control was 95.2%. Responses were ongoing at the time of data cut-off in two patients with CR and one patient with PR (figure 2B, C). Analysis of 27 response-evaluable patients found that the PD-L1 expression level could not be used as a biomarker for predicting clinical response (p=0.395; online supplementary figure S1A). Whole-exome sequencing was performed on patients’ biopsied tumor samples and paired peripheral blood mononuclear cells, which were allocated to a responding group (CR+PR) and non-responding group (SD+PD) according to the clinical response. TMB and TNB were generally low in this study (online supplementary figure S2). However, the median value of TMB, TNB, and fitness was higher in the responding group than in the non-responding group, while the median value of heterogeneity was lower in the responding group; of these differences, fitness had statistical difference (p=0.041; figure 3A). Mutations of RYR2, MUC4, and APOB were detected only in samples from the responding group (figure 3B). We performed exploratory analysis to study the association between the activation of peripheral T cells and clinical antitumor activity. The evaluation of T cells in peripheral blood showed that the baseline percentage of CD3+ cells in responders was higher than that in non-responders (p=0.046; online supplementary figure S3A). The proportion of HLA-DR+ CD3+ cells in patients’ peripheral blood increased after the start of the combination therapy, especially in patients with an objective response (p=0.009). However, a statistical difference was not observed between responders and non-responders (online supplementary figure S3B, C). The association between changes in peripheral serum cytokines and chemokines at baseline (C1D0) and the day before the first dose of the third cycle (C3D0) and clinical response was also assessed. The concentrations of serum sFasL and granzyme A were higher in non-responders than in responders after two cycles of combination treatment (p=0.042 and p=0.048, respectively), while the concentrations of IL-2, IL-18, sFasL and CCL2 dropped significantly in responders compared with non-responders (p=0.036, p=0.047, p=0.012 and p=0.042, respectively; online supplementary figure S4A, B).

10.1136/jitc-2019-000367.supp2 Supplementary data

**Table 3:** Clinical antitumor activity

	Overall (n=27)	Cohort A (n=6)	Cohort B (n=21)
Confirmed objective response	15 (55.6)	2 (33.3)	13 (61.9)
Best overall response			
Complete response	5 (18.6)	1 (16.7)	4 (19.0)
Partial response	10 (37)	1 (16.7)	9 (42.9)
Stable disease	10 (37)	3 (50.0)	7 (33.3)
Progressive disease	2 (7.4)	1 (16.7)	1 (4.8)
Disease control	25 (92.6)	5 (83.3)	20 (95.2)

Data are n (%), unless otherwise specified.

Responses were assessed in accordance with the Response Evaluation Criteria in Solid Tumors version 1.1.

### PFS and biomarkers

The median PFS in this study was 6.1 months (95% CI 3.4 to 8.2), and the proportions of patients who were progression-free at 6 months and 12 months were 51.9% (95% CI 31.9 to 68.6) and 18.5% (95% CI 6.8 to 34.8), respectively (figure 2D). A comparison between cohort A and cohort B showed that chemotherapy-naive patients could obtain longer median PFS than those who had received chemotherapy; however, there was no statistical difference. Further analysis found that patients who were administered more than four cycles of combination treatment had longer PFS than those who received four cycles or less (p=0.024), and PD-L1 expression status could not be established as a biomarker for predicting PFS (p=0.125; online supplementary figure S1B). We also analyzed the impact of TMB, TNB, and fitness on PFS in this study. However, there was no correlation between the above biomarkers and PFS (figure 3C, online supplementary figure S5). Analysis of peripheral serum cytokines found that patients whose concentration of IFN-γ decreased following combination therapy could obtain longer PFS than those whose concentration of IFN-γ increased or remained the same after treatment (p=0.033; figure 3D), and a similar association was found between the decrease in MCP-1 and PFS (p=0.019; figure 3D).

### OS and biomarkers

The median OS was 8.5 months (95% CI 5.0 to 12.5), and the 12-month OS rate and 18-month OS rate were 33.3% (95% CI 16.8 to 50.9) and 24.7% (95% CI 10.2 to 42.4), respectively (figure 2D). There was no statistical difference between the median OS of cohort A and that of cohort B. Four cycles or more of combination therapy was a parameter that could be correlated with longer OS (HR 0.595 (95% CI 0.398 to 0.89), p=0.012; online supplementary figure S1C), while a correlation between PD-L1 expression and OS was not established (p=0.499; online supplementary figure S1C). Whole-exome sequencing results showed that a 1.37 neoantigens/Mb cut-off value, as used for the TNB in this study, had prognostic value, and patients with TNB greater than 1.37 neoantigens/Mb had significantly longer OS than those with TNB of 1.37 neoantigens/Mb or fewer (p=0.048; figure 3C). Analysis of serum cytokines detected that the concentration of sFasL and IFN-γ dropped significantly in patients with longer OS (p=0.00076, p=0.032; figure 3E). The changes in granulysin, MCP-1, IL-17a, IL-23, TNF-α and granzyme B in serum following combination therapy had no statistical influence on OS (figure 3E; online supplementary figure S6).

## Discussion

We assessed the efficacy and safety of nivolumab in combination with gemcitabine and cisplatin in patients with advanced BTCs in this study. The most frequent, especially severe adverse events in this study, were hematological toxicities, which were mainly attributed to the chemotherapy. However, we observed that the incidence of grade 3 or higher thrombocytopenia was much higher in this study than that currently reported for gemcitabine and cisplatin chemotherapy,^4 5 7^ and whether the addition of nivolumab to chemotherapy affected thrombocytopenia remains unclear. Indeed, thrombocytopenia is a common toxicity of ICIs.^18–20^ One study reported that the number of PD-L1-expressing platelets was diminished in the blood of four patients with lung cancer treated with the anti-PD-L1 antibody atezolizumab within the first 7 days of therapy.^21^ Another study reported that the average time to onset of thrombocytopenia induced by ICIs was 70 days, and the average platelet count was 61×10^9^/L, with an average decrease of 70% from baseline.^19^

Gemcitabine and cisplatin or oxaliplatin are the standard therapies for advanced BTCs; however, only 26% of patients respond to this chemotherapy regimen at most, accompanied by a no more than 8-month median PFS and an approximately 12-month median OS.^4 5^ Whether ICIs alone or in combination with standard chemotherapy have the potential to improve the response rate and prognosis of advanced BTCs is uncertain. Several studies with small sample sizes evaluated the value of ICIs in treating advanced BTCs.^12 13 22^ Nevertheless, the effectiveness of ICIs deserves further assessment, including an assessment of the capability of ICIs to reverse chemotherapy resistance. Cohort A in this study enrolled seven patients who were previously treated with gemcitabine-based or cisplatin-based chemotherapy, and one obtained CR, while one obtained PR, indicating that nivolumab was capable of resensitizing gemcitabine and cisplatin chemotherapy. Compared with those reported historically, this study found better tumor shrinkage and disease control with the combination of nivolumab and cisplatin plus gemcitabine in chemotherapy-naive patients.^4–6^ The improvement of clinical response may be due to the synergistic interaction between chemotherapy and ICIs, in which gemcitabine reduced the amount of circulating myeloid derived suppressor cells (MDSCs), favoring the reprogramming of tumor associated macrophages (TAMs) toward an immunostimulatory phenotype, boosting cross-priming and increasing the antigenicity of cancer cells,^23 24^ and ICIs in return neutralized the unwarranted immunosuppressive effects of anticancer drugs and maximized the immunostimulatory effects of chemotherapy.^15^ The immunostimulatory potential of gemcitabine has been identified in experimental tumor models with combinations including ipilimumab and in patients with metastatic solid tumors when combined with adoptive cell transfer therapy.^25 26^

Although PD-L1 expression as a biomarker in predicting the efficacy of ICIs has been extensively studied in various types of cancers,^27–29^ contradictory results have indicated that PD-L1 expression remains an imperfect predictor, as some studies established a positive correlation between PD-L1 and ICI response, while others detected no association.^30 31^ Our data found that the efficacy of nivolumab in combination with gemcitabine and cisplatin was independent of PD-L1 expression level. We also evaluated the potential of other biomarkers in predicting the response to nivolumab in combination with gemcitabine and cisplatin, including TMB, TNB, and fitness, which are current factors of high interest for predicting the clinical response to ICI monotherapy or ICI combination with chemotherapy.^17 32–37^ Despite the lack of statistical significance, which was probably caused by the limited sample size, we observed that higher TMB and TNB and lower heterogeneity may result in a better clinical response, suggesting that these factors may be potent biomarkers for predicting response. Recently, there has been growing interest in developing blood-derived or serum-derived predictive biomarkers of ICI response across a variety of cancer types,^38–40^ especially on-therapy biomarkers.^41^ We analyzed the early on-therapy change in peripheral serum cytokines and circulating T cell levels and found that a higher percentage of baseline CD3+ cells and a decrease in IL-2, IL-18, sFasL and CCL2 levels in peripheral blood could predict a better outcome of ICI-based combination therapy.

Despite exciting data related to clinical response, the survival data in this study, such as median PFS, PFS at 6 months, OS, and OS at 12 months, were disappointing and no significant survival benefit was found when compared with the data achieved by chemotherapy alone in the UK-ABC-02 and BINGO trials.^4 5^ One possible cause was the high incidence of grade 3–4 hematological toxicities, which resulted in dose reduction of the study drugs or treatment suspension. We assessed the correlation between prognostic biomarkers and the chemotherapy and immunotherapy combination and found that patients with higher pretreatment TNB seemed to have better OS than those with a low pretreatment TNB. Disappointingly, correlations between other prognostic biomarkers, including PD-L1 expression level, and OS were not established in this study. However, low concentrations of T helper 1 (Th1)-type cytokines and cytolytic enzymes such as IFN-γ were detected in patients with longer PFS and OS, which seemed to contradict the general pattern. This might partly be explained by the sample type and sampling time. An increase in IFN-γ is often observed in CD8+ T cells after cancer immunotherapy, but the serum IFN-γ signature had no correlation with the OS of patients with squamous cell lung carcinoma.^42^ Moreover, elevated expression of serum IFN-γ was detected in the early post-treatment stage of immunotherapy, but no significant difference was noted at the late post-treatment stage (days 50–120), similar to our sampling time (post two cycles).^43 44^ Meanwhile, chemotherapy has been proven to attenuate the expression of proinflammatory cytokines.^45^

Our study may be susceptible to research bias due to its non-randomized design. A larger randomized trial is needed to confirm the results of this preliminary study on the activity of nivolumab in combination with chemotherapy in BTCs. In summary, our study suggested that nivolumab in combination with gemcitabine and cisplatin had promising antitumor efficacy and a manageable safety profile in advanced unresectable or metastatic BTCs, providing a potential treatment option and supporting further study of this combination therapy in patients with this cancer.
