# Supplementary material for: Efficacy and biomarker analysis of nivolumab plus gemcitabine and cisplatin in patients with unresectable or metastatic biliary tract cancers: results from a phase II study
Source: J Immunother Cancer. 2020 Jun 2;8(1):e000367. doi: 10.1136/jitc-2019-000367 (PMC7269541; doi:10.1136/jitc-2019-000367)
Supplement: Supplementary data [file jitc-2019-000367supp001.pdf]

The following protocol information is provided solely to describe how the authors conducted the research underlying the published report associated with the following article:

Efficacy analysis of nivolumab in combination with gemcitabine and cisplatin in patients with unresectable or metastatic biliary tract cancers

Feng K, *et al. J Immunother Cancer* 2020; 8:e000367. doi: 10.1136/jitc-2019-000367

TABLE OF CONTENTS

TITLE PAGE…………………………………………………………………...5

SYNOPSIS………………………………………………………………………7

1 INTRODUCTION AND STUDY RATIONALE……………………….....12

1.1 Biliary tract cancers……………………………………………………….. 12

1.2 Background and rationale for conducting this study…………………………….12

1.3 Rationale for study design……………………………………………….…14

1.4 Research Hypothesis…………………………………………………......15

1.5 Objectives…………………………………………………………………..16

1.6 Overall Risk/Benefit Assessment……………………………………….…16

2 ETHICAL CONSIDERATIONS…………………………………… …17

2.1 Ethical conduct of the study……………………………………………...17

2.2 Institutional Review Board/Independent Ethics Committee……………………..17

2.3 Subjects data protection…………………………………………………….18

2.4 Informed Consent…………………………………………………………...18

2.5 Change to the protocol and informed consent form…………………………….. 20

2.6 Audits and inspections…………………………………………………….. 20

3 INVESTIGATIONAL PLAN…………………………………………….. 21

3.1 Study design and duration……………………………………………….…21

3.2 Study population…………………………………………………………….22

3.3 Concomitant treatments……………………………………………………….25

3.4 Discontinuation of investigational product……………………………………25

4 TREATMENTS………………………………………………………..…26

4.1 Investigational Products and Non-investigational Products……………………...26

4.2 Handling and Dispensing……………………………………………….…..27

4.3 Preparation and Administration…………………………………………..…27

4.4 Dose reduction or Discontinuation of study drugs………………………………27

5 ADVERSE EVENTS AND CLINICAL ADMINISTRATION………... 29

5.1 Definition of adverse events………………………………………………...29

5.2 Definitions of serious AE…………………………………………………..29

5.3 Recording of AEs…………………………………………………………..30

5.4 Collected information for AEs……………………………………………..30

5.5 AEs based on signs and symptoms……………………………………….31

5.6 AEs based on examination and tests……………………………………..32

5.7 Disease progression…………………………………………………...…32

5.8 The report of deaths………………………………………………………...32

5.9 Management of the treatment related toxicities…………………………….33

6 STATISTICAL ANALYSES OF TREATMENT……………………...33

6.1 Statistical considerations………………………………………………....33

6.2 Sample size estimate……………………………………………………..34

6.3 Outcome measures for analysis……………………………………….....34

6.4 Safety Assessments……………………………………………………....35

6.5 Tumor PD-L1/PD-L2 multiplex immunofluorescence assessment…………...…35

6.6 Peripheral blood T cell assessment………………………………...…37

Feng K, *et al. J Immunother Cancer* 2020; 8:e000367. doi: 10.1136/jitc-2019-000367

6.7 Methods for statistical analyses……………………………………………………37

7 LIST OF ABBREVIATIONS…………………………………………..…...39

8 REFERENCES………………………………………………………….....42

APPENDIX 1 Response Evaluation Criteria in Solid Tumors……………....46

APPENDIX 2 COMMON TERMINOLOGY CRITERIA FOR ADVERSE EVENTS V5.0 (CTCAE)………………………………………………..........48

APPENDIX 3 ECOG Performance Status Scale…………………………...49

APPENDIX 4 New York Heart Association (NYHA) Classification………50

Chinese PLA General Hospital

Title Page

Efficacy analysis of nivolumab in combination with gemcitabine and cisplatin in patients with unresectable or metastatic biliary tract cancers

**Principal Investigator**	Weidong Han PhD<br>Bio-therapeutic Department<br>Molecular & Immunological Department<br>Chinese PLA General Hospital<br>Beijing 100853 China
**Co-Investigator**	Kaichao Feng MD<br>Qian Mei PhD<br>Yang Liu MD<br>Qingming Yang MD<br>Liang Dong BA<br>Jiejie Liu BA<br>Xiang Li BA
**Regulatory Sponsor**	Weidong Han PhD<br>Bio-therapeutic Department<br>Molecular & Immunological Department<br>Chinese PLA General Hospital<br>Beijing 100853 China

**Funding Sponsor**	National Natural Science Foundation of China<br>National Key Research and Development Program of China
**Study Product**	Nivolumab; Gemcitabine; Cisplatin
**Original Protocol**	April 1, 2017

CONFIDENTIAL

This document is confidential and the property of Chinese PLA General Hospital. No part of it may be transmitted, reproduced, published, or used by other persons without prior written authorization from the study sponsor.

STUDY TEAM

<u>Clinical Trial Site-Chinese PLA General Hospital</u>

Principal Investigator
Weidong Han PhD

Clinical Team
Kaichao Feng MD
Yang Liu MD
Qingming Yang MD

Laboratory Team
Qian Mei PhD
Liang Dong BA
Jiejie Liu BA
Xiang Li BA

Statistical Support
Qian Mei PhD

Feng K, *et al. J Immunother Cancer* 2020; 8:e000367. doi: 10.1136/jitc-2019-000367

SYNOPSIS

Protocol Title: Efficacy analysis of nivolumab in combination with gemcitabine and cisplatin in patients with unresectable or metastatic biliary tract cancers

Investigational Product(s), Dose and Mode of Administration, Duration of Treatment with Investigational Product(s): Gemcitabine dosed at 1000mg/m^2 on day 1 and day 5, cisplatin dosed at 75mg/m^2 on day 1, and nivolumab dosed at 3mg/kg on day 3 were given intravenously every 3 weeks for a maximum of 6 cycles. Patients who responded to combination treatment or stable disease will switch to maintenance therapy with nivolumab alone or in combination with gemcitabine every six to eight weeks until disease progression, intolerable toxicity, death, withdrawal of consent or investigator decision.

Study Phase: II

Research Hypothesis: The combination of nivolumab with standard gemcitabine and cisplatin chemotherapy could improve the overall response rate and survival of patients with advanced BTCs when compared with gemcitabine and cisplatin alone without aggravation of toxicities.

Study Objectives:

Primary Objective: To evaluate the overall response rate (ORR) of nivolumab in combination with gemcitabine and cisplatin chemotherapy for advanced unresectable or metastatic BTCs.

Secondary Objectives: To evaluate safety and other parameters of clinical efficacy of nivolumab plus gemcitabine and cisplatin in subjects with advanced BTCs with other various parameters, including progression free survival (PFS) at 6 months, disease control rate (DCR), overall survival (OS).

Exploratory Objectives: To evaluate pathological, immunological or clinical predictive factors for response and toxicity.

Study Design: Subjects will receive study drug as detailed in Figure 1

Figure 1:

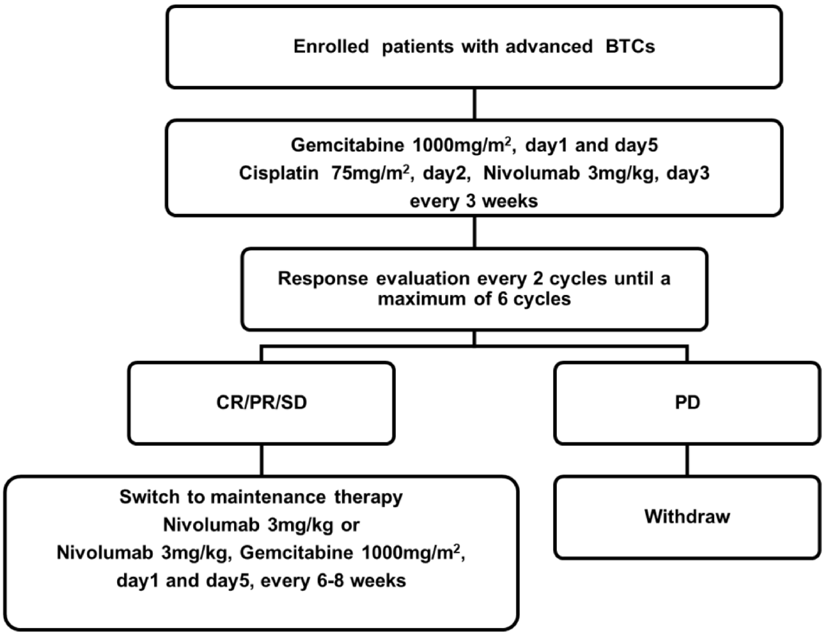

Study Population:

Eligible subjects should meet all criteria of inclusion and exclusion, details as follows：

Inclusion Criteria:

1. Age from 18 to 75 years with estimated life expectancy >3 months.

2. Histopathological confirmed advanced unresectable or metastatic BTCs and had at least one measurable disease (≥1cm).

3. Subjects should provide fresh tumor tissue samples or formalin-fixed paraffin embedded tumor archival samples within 3 months and willing to accept tumor re-biopsy in the process of the study.

4. Subjects may have received prior radiotherapy, chemotherapy, or other local ablative therapies, which completed ≥ 4 weeks prior to registration AND patient has recovered to <= grade 1 toxicity.

5. ECOG (Eastern Cooperative Oncology Group) performance status of 0-2.

6. Adequate organ and marrow function obtained ≤ 2 weeks of treatment initiation as defined below:

Leukocytes greater than or equal to 3.0 x 10^9/L.

Absolute neutrophil count greater than or equal to 1.0 x 10^9/L.

Platelets greater than or equal to 100 x 10^9/L.

Hemoglobin greater than or equal to 90 g/L.

Total bilirubin less than or equal to 2 x ULN.

Serum albumin should be no less than 25g/L.

ALT or AST less than 2 x ULN.

Measured creatinine clearance ≥60 mL per min.

7. Ability to understand and willingness to sign a written informed consent document.

8. Women of child-bearing potential and men must agree to use adequate contraception (hormonal or barrier method of birth control; abstinence) prior to study entry, and up to 120 days after the last dose of the drug.

Exclusion Criteria:

1. Active, known or suspected autoimmune diseases.

2. Known brain metastases or active central nervous system (CNS). If patients with CNS metastases were treated with radiotherapy for at least 3 months prior to enrollment and have no central nervous symptoms and are off corticosteroids, they will be eligible but will need a brain MRI prior to enrollment.

3. Subjects are being treated with either corticosteroids (>10 mg daily prednisone equivalent) or other immunosuppressive medications within 14 days of enrollment.

4. Prior therapy with anti-PD-1, anti-PD-L1, or anti-CTLA-4 antibody (including Ipilimumab or any other antibody specifically targeting T-cell co-stimulation or checkpoint pathways).

5. History of severe hypersensitive reactions to other monoclonal antibodies.

6. History of allergy or intolerance to study drug components.

7. Substance abuse, medical, psychological or social conditions that may interfere with the patient's participation in the study or evaluation of the study results.

8. History or concurrent condition of interstitial lung disease of any grade or severely impaired pulmonary function.

9. Uncontrolled intercurrent illness, including ongoing or active systemic infection, symptomatic congestive heart failure, unstable angina pectoris, cardiac arrhythmia (excluding insignificant sinus bradycardia and sinus tachycardia) or psychiatric illness/social situations and any other illness that would limit compliance with study requirements and jeopardize the safety of the patient.

10. History of human immunodeficiency virus (HIV) infection or acquired immunodeficiency syndrome (AIDS).

11. Pregnant or breast-feeding. Women of childbearing potential must have a pregnancy test performed within 7 days before the enrollment, and a negative result must be documented.

12. Previous or concurrent cancer within 3 years prior to treatment start EXCEPT for curatively treated cervical cancer in situ, non-melanoma skin cancer, superficial bladder tumors [Ta (non-invasive tumor), Tis (carcinoma in situ) and T1 (tumor invades lamina propria)].

13. Vaccination within 30 days of study enrollment.

14. Active bleeding or known hemorrhagic tendency.

15. Subjects with unhealed surgical wounds for more than 30 days.

16. Being participating any other trials or withdraw within 4 weeks.

Study Assessments:

Safety assessments: Adverse events will be assessed documented and graded using National Cancer Institute Common Terminology Criteria for Adverse Events (CTCAE) Version 5.0. ALL subjects will be followed up for adverse events up to 120 days after the last dose of study drugs

Efficacy assessments: CT with contrast or MRI will be used to assess the antitumor efficacy according to Response Evaluation Criteria in Solid Tumors (version 1.1). Positron Emission Tomography-Computed Tomography (PET-CT) will be used to confirm the response status when CT with contrast or MRI show unconfirmed complete response.

Potential response and toxicity biomarkers: Serial blood samples will be collected in the study and tumor re-biopsy will be encouraged when subjects being evaluated as responsive or progressive disease. The peripheral T cell phenotype and activity will be detected by FACS, and tumor PD-L1 levels will be assessed by multiplex immunostaining.

Endpoints:

Primary endpoints: ORR

Secondary endpoints: DCR, Safety, PFS at 6 months, OS.

Exploratory endpoints: pathological, immunological or clinical predictive biomarkers for response and prognosis.

Feng K, *et al. J Immunother Cancer* 2020; 8:e000367. doi: 10.1136/jitc-2019-000367

1 INTRODUCTION AND STUDY RATIONALE

1.1 Biliary tract cancers

Biliary tract cancers (BTCs) represent a diverse group of highly invasive heterogeneous epithelial cancers arising from the biliary tract. Base on their anatomic location, BTCs are classified into gallbladder carcinoma (GBCA), intrahepatic cholangiocarcinoma (iCCA), perihilar cholangiocarcinoma (pCCA) and distal cholangiocarcinoma (dCCA). The incidence of BTCs has increased globally over the past few decades[1], with reported prevalence of 169,000 patients diagnosed with BTCs in 2016[2]. Surgical resection is potentially curative treatment option for early-stage BTCs, however, most patients with BTCs already have locally advanced or metastatic disease at the time of diagnosis. Even in cases of resection, recurrence is seen in > 60% of patients within the first or the second year[3].

For patients with advanced unresectable and/or metastatic BTCs, chemotherapy is still the main systemic therapy. Gemcitabine in combination with cisplatin was reported achieving a 11.7-month median overall survival and thus were recommended as the standard first-line systemic therapy[4]. Other chemotherapeutic regimens such as gemcitabine and oxaliplatin with or without cetuximab[5], capecitabine plus cisplatin[6], were also tested and showed similar efficacy when compared with gemcitabine and cisplatin as first-line chemotherapy for advanced biliary tract cancers. Recently, there are several trials of small molecule kinase inhibitors in advanced biliary tract cancers by targeting FGFR, IDH, MET, Mesothelin, BRCA and other mutated genes, however, the results are disappointing[7].

Feng K, et al. J Immunother Cancer 2020; 8:e000367. doi: 10.1136/jitc-2019-000367

1.2 Background and rationale for conducting this study

In recent years, immune checkpoint inhibitors, exemplified by antibodies that target programmed cell death-1 (PD-1) and programmed cell death-ligand 1 (PD-L1), have been studied across a variety of tumors and demonstrated robust and durable anti-tumor activity, coupled with low rates of immune-mediated toxicity[8-9]. Preclinical data have suggested an encouraging future for targeting checkpoint pathways in biliary tract tumors. Multiple studies using immunohistochemistry have observed PD1/PDL1 expression in neoplastic cells and inflammatory cell aggregates in cases of ICC. Gani et al reported PD-L1 expression on cells in the tumor front in 72% of samples of resected ICC. This PD-L1 expression was significantly associated with 60% reduction in OS compared to PD-L1 negative counterparts[10]. Sabbatino et al reported PD1 and PD-L1 expression in 100% of resected ICC specimen and evidence of antitumor T-cell mediated immune responses[11]. Transcriptome sequencing and clustering of gene-expression profiles revealed a subgroup of patients with biliary tract cancers with a high mutational load, resulting in abundant tumor-specific neoantigens, and enrichment for expression of immune-related genes[12].

Currently, clinical data with immune checkpoint inhibitors in biliary tract cancers are very limited. Interim safety and efficacy data from KEYNOTE-028 basket trial reported that a total of 24 patients with PD-L1 positive biliary tract cancers were enrolled in this study. Of the 24 patients, 4 (17.4%) obtained a partial response, 4 had stable disease, and 12 had disease progression[13]. In another basket trial, PD-1 blockade with pembrolizumab resulted in 100% disease control rate in 4 patients with tumor DNA mismatch repair (MMR)-deficient cholangiocarcinoma (one of the patients had a complete response, and the other three had stable disease)[14].

Although MMR deficiency and/or microsatellite instability (MSI) is associated with higher

response rates and durability of responses to immune-checkpoint blockade, MMR deficiency has been reported to occur in 5–10% of biliary tract cancers[15], limiting the clinical use of immune checkpoint inhibitors in biliary tract cancers. Meanwhile, Interim analysis of KEYNOTE-028 basket trial revealed that the sensitivity of PD-L1 positive biliary tract cancers to immune checkpoint inhibitors was low. Thus, strategies that could improve the efficacy of immune checkpoint inhibitors are needed.

Clinician oncologists often argue that conventional chemotherapeutics cannot have positive effects on anticancer immune responses. However, recent studies illustrated that routine dose chemotherapy could allow for the elicitation of perfectly normal immune responses by vaccines against the influenza virus[16]. In line with this notion, experimental DC-, DNA-, or peptide-based anticancer vaccines can elicit tumor-targeting immune responses in patients treated with conventional chemotherapeutics[17]. In return, immunotherapy could neutralize the unwarranted immunosuppressive effects of anticancer drugs and can be harnessed to maximize the immunostimulatory effects of chemotherapy[18].Therefore, chemotherapy have the potential to interact positively with ICB-based immunotherapy.

1.3 Rationale for study design

Nivolumab

Nivolumab, a genetically engineered, fully human, IgG4 immune checkpoint inhibitor antibody manufactured by Bristol-Myers Squibb, contains an engineered hinge region mutation (S228P) designed to prevent exchange of IgG4 molecules. It binds PD-1 on activated immune cells with high affinity (K_D=2.6 nmol/L by Scatchard analysis to polyclonally activated human T cells) to block

PD-1 interaction with PD-L1 and PD-L2 ligands, thereby attenuating inhibitory signals and augmenting the host anti-tumor response[19]. Up to now, nivolumab is approved by US FDA for various indications, including unresectable or metastatic melanoma, metastatic non-small cell lung cancer, advanced renal cell carcinoma, and so on.

Gemcitabine

Gemcitabine, a nucleoside analog used commonly for the treatment of pancreatic carcinoma, lung squamous carcinoma, and other cancers, can reduce the amount of circulating MDSCs, favors the reprogramming of TAMs toward an immunostimulatory phenotype, and boosts cross-priming[20]. Besides the direct immunostimulatory effects, gemcitabine stimulates the expression of MHC class I molecules by cancer cells, thereby increasing their antigenicity[21].

Cisplatin

Cisplatin, a commonly used cytotoxic chemotherapeutic drug, was reported having the potential to enhance immune-stimulating activity in a dose-dependent manner by reducing expression of the T cell inhibitory molecule programmed death receptor-ligand 2 (PD-L2) on both human DCs and human tumor cell, resulting in enhanced antigen-specific proliferation and Th1 cytokine secretion as well as enhanced recognition of tumor cells by T cells[22]. The combination of cisplatin and gemcitabine could significantly reduce the percentage of regulatory T cells in non-small cell lung cancer[23].

1.4 Research Hypothesis

The combination of nivolumab with standard gemcitabine and cisplatin chemotherapy could improve the overall response rate and survival of patients with advanced BTCs when compared with

gemcitabine and cisplatin alone without aggravation of toxicities.

1.5 Objectives

1.5.1 Primary Objective

To evaluate the overall response rate (ORR) of nivolumab in combination with gemcitabine and cisplatin for advanced unresectable or metastatic BTCs.

1.5.2 Secondary Objectives:

To evaluate safety and other efficacy parameters, including the disease control rate (DCR), progression free survival (PFS), PFS at 6 months, overall survival (OS)

1.5.3 Exploratory Objectives

To evaluate pathological, immunological or clinical predictive factors for response and toxicity.

1.6 Overall Risk/Benefit Assessment

Biliary tract cancers are highly invasive heterogeneous epithelial with the poorest prognosis. Approximately 90% of the patients with biliary tract cancers were diagnosed with advanced unresectable or metastatic status, resulting their median overall survival (OS) rarely exceeding 6-8 months. Even in cases of resection, recurrence is seen in > 60% of patients within the first or the second year. However, current systemic strategy for care is gemcitabine-based chemotherapy, which provide limited benefit for patients with advanced biliary tract cancers.

Feng K, *et al. J Immunother Cancer* 2020; 8:e000367. doi: 10.1136/jitc-2019-000367

2 ETHICAL CONSIDERATIONS

2.1 Ethical conduct of the study

This study will be performed in accordance with Good Clinical Practice (GCP), as defined by the International Conference on Harmonization (ICH) and in accordance with the ethical principles underlying the policy of Chinese PLA General Hospital on Bioethics and Human Biological Samples.

The study will be conducted in compliance with the protocol. The protocol and any amendments and the subject informed consent will receive the approval/ favorable opinion from Institutional Review Board/Independent Ethics Committee (IRB/IEC) prior to initiation of the study.

Personnel involved in this study will be qualified by education, training, and experience to perform their respective tasks.

2.2 Institutional Review Board/Independent Ethics Committee

As Ethics Committee should approve the final study protocol, including the final version of the Informed Consent form and any other written information and materials to be provided to the subjects. The investigators should also provide the IRB/IEC with a copy of the Investigator Brochure or product labeling information to be provided to subjects and any updates.

The opinion of the Ethics Committee should be given in written. The investigators should submit the written approval to Chinese PLA General Hospital before enrollment of any subject into the study.

The Ethics Committee should approve all advertising used to recruit subjects for the study.

The research team should approve any modification to the Informed Consent form that are

needed to meet local requirements.

If required by local regulation, the protocol should be re-approved by the Ethics Committee annually.

Before enrollment of any subject into the study, the final study protocol, including the final version of the Informed Consent form, should be approved by the national regulatory authority or a notification to the national regulatory authority is done, according to local regulations.

Each principal investigator is responsible for providing the Ethics Committee with reports of any serious or unexpected adverse drug reactions from any other study conducted with the investigational product. The research team will provide this information to the principal investigator so that he/she can meet the reporting requirements.

2.3 Subjects data protection

The Informed Consent Form will incorporate wording that complies with relevant data protection and privacy legislation.

Precautions are taken to preserve confidentiality and prevent data being linked to the identity of the subjects. In exceptional circumstances, however, certain individual might see both the data and the personal identifiers of a subject. Regulatory authorities may require assess to the relevant files, though the subject’s medical information and the genetic files would remain physically separate.

2.4 Informed Consent

Investigators must ensure that subjects are clearly and fully informed about the purpose, potential risks, and other critical issues regarding clinical studies in which they volunteer to

participate.

In situations where consent cannot be given to subjects, their legally acceptable representatives are clearly and fully informed about the purpose, potential risks, and other critical issues regarding clinical studies in which the subject volunteers to participate.

Investigators will provide an appropriate (ie, Global or Local) sample informed consent form which will include all elements required by ICH, GCP and applicable regulatory requirements. The sample informed consent form will adhere to the ethical principles that have their origin in the Declaration of Helsinki.

Investigators must: 1) Provide a copy of the consent form and written information about the study in the language in which the subject is most proficient prior to clinical study participation. The language must be non-technical and easily understood. 2) Allow time necessary for subject or subject's legally acceptable representative to inquire about the details of the study. 3) Obtain an informed consent signed and personally dated by the subject or the subject's legally acceptable representative and by the person who conducted the informed consent discussion. 4) Obtain the IRB/IEC's written approval/favorable opinion of the written informed consent form and any other information to be provided to the subjects, prior to the beginning of the study, and after any revisions are completed for new information. 5) If informed consent is initially given by a subject's legally acceptable representative or legal guardian, and the subject subsequently becomes capable of making and communicating his or her informed consent during the study, consent must additionally be obtained from the subject. 6) Revise the informed consent whenever important new information becomes available that is relevant to the subject's consent. The investigator, or a person designated by the investigator, should fully inform the subject or the subject's legally acceptable representative

or legal guardian, of all pertinent aspects of the study and of any new information relevant to the subject's willingness to continue participation in the study. This communication should be documented.

The rights, safety, and well-being of the study subjects are the most important considerations and should prevail over interests of science and society.

2.5 Change to the protocol and informed consent form

If there are any substantial changes to the study protocol, then these changes will be documented in study protocol amendment and where required in a new version of the study protocol.

The amendment should be approved by the relevant Ethics Committee and if applicable, also by the national regulatory authority before implementation. Local requirements are to be followed for revised protocols.

If local regulations require, any administrative change will be communicated to or approved by each Ethics Committee.

2.6 Audits and inspections

Ethics Committee perform audits or inspections at the center, including source data verification. The purpose of an audit or inspection is to systematically and independently examine all study-related activities and documents, to determine whether these activities were conducted, and data were recorded, analyzed, and accurately reported according to the protocol, Good Clinical Practice (GCP), guideline of the International Conference on Harmonisation (ICH), and any applicable regulatory requirements.

3 INVESTIGATIONAL PLAN

3.1 Study design and duration

This is a Phase II open-label, single-center, single-arm study of Nivolumab, an anti-PD-1 antibody, in combination with Gemcitabine and cisplatin for advanced unresectable or metastatic Biliary tract cancers. Primary Objective: To evaluate the overall response rate (ORR) of nivolumab in combination with gemcitabine and cisplatin chemotherapy for advanced unresectable or metastatic BTCs. Secondary Objectives: To evaluate safety and other parameters of clinical efficacy of nivolumab plus gemcitabine and cisplatin in subjects with advanced BTCs with other various parameters, including progression free survival (PFS) at 6 months, disease control rate (DCR), overall survival (OS). The exploratory objectives are to evaluate pathological, immunological or clinical predictive factors for response and toxicity.

This study includes sections of Screening, Treatment, and Follow-up. Patients receive treatment until disease progression, death, unacceptable toxicity, or withdrawal of consent. After receiving a maximum of 6 cycles of combination treatment, responsive patients (CR, PR and durable SD) will switch to maintenance therapy. The response assessment will be performed every 2 cycles by CT with contrast or MRI. PET-CT will be required when CT with contrast or MRI showed unconfirmed complete response. The toxicity assessment following administration of the treatment will also be obtained.

Screening:

a. Begins by establishing subject's initial eligibility and signing of the informed consent form; Subject is enrolled via outpatient service and obtains a subject ID.

b. Tumor tissues (archival or fresh) was suggested to obtain as possible for exploratory study.

Feng K, *et al. J Immunother Cancer* 2020; 8:e000367. doi: 10.1136/jitc-2019-000367

c. Baseline disease, treatment history and tumor assessments should be performed within 2 weeks.

d. Subjects are assessed for study eligibility within the required timeframe.

Treatment:

a. To characterize safety and efficacy of nivolumab in combination of gemcitabine and cisplatin.

b. Treatment should be administrated within 1 week of enrollment.

c. Clinical examinations and biochemistry tests will be collected before the start of each cycle treatment. Adverse events should be documented every cycle of treatment.

d. Samples of peripheral blood will be collected every cycle of treatment for T cell function testing.

e. Subjects who are responsive or resistant to the treatment will be suggested to do re-biopsy of target lesions.

f. All study drugs will be administered intravenously in each cycle until disease progression, serious toxicity, withdrawal of consent.

g. Subjects will be evaluated for response by investigators per immune-related response criteria.

Follow-up:

a. Safety follow-up will be performed up to 120 days after the last dose of study drugs.

b. Subjects will be followed for PFS and OS every 3 month until death.

c. Subjects who discontinue the study for reasons other than disease progression will be required to continue radiographic assessments every 3 months until death or withdrawal of study consent.

Feng K, *et al. J Immunother Cancer* 2020; 8:e000367. doi: 10.1136/jitc-2019-000367

3.2 Study population

Subjects must meet the following criteria for entry into this study.

Inclusion criteria:

1. Age from 18 to 75 years with estimated life expectancy >3 months.

2. Histopathological confirmed advanced unresectable or metastatic BTCs and had at least one measurable disease (⩾1cm).

3. Subjects should provide fresh tumor tissue samples or formalin-fixed paraffin embedded tumor archival samples within 3 months and willing to accept tumor re-biopsy in the process of the study.

4. Subjects may have received prior radiotherapy, chemotherapy, or other local ablative therapies, which completed ≥ 4 weeks prior to registration AND patient has recovered to <= grade 1 toxicity.

5. ECOG (Eastern Cooperative Oncology Group) performance status of 0-2.

6. Adequate organ and marrow function obtained ⩽ 2 weeks of treatment initiation as defined below:

Leukocytes greater than or equal to 3.0 x 10^9/L.

Absolute neutrophil count greater than or equal to 1.0 x 10^9/L.

Platelets greater than or equal to 100 x 10^9/L.

Hemoglobin greater than or equal to 90 g/L.

Total bilirubin less than or equal to 2 x ULN.

Serum albumin should be no less than 25g/L.

ALT or AST less than 2 x ULN.

Measured creatinine clearance ≥60 mL per min.

7.Ability to understand and willingness to sign a written informed consent document.

8.Women of child-bearing potential and men must agree to use adequate contraception (hormonal or barrier method of birth control; abstinence) prior to study entry, and up to 120 days after the last dose of the drug.

Exclusion criteria:

1. Active, known or suspected autoimmune diseases.

2. Known brain metastases or active central nervous system (CNS). If patients with CNS metastases were treated with radiotherapy for at least 3 months prior to enrollment and have no central nervous symptoms and are off corticosteroids, they will be eligible but will need a brain MRI prior to enrollment.

3. Subjects are being treated with either corticosteroids (>10 mg daily prednisone equivalent) or other immunosuppressive medications within 14 days of enrollment.

4. Prior therapy with anti-PD-1, anti-PD-L1, or anti-CTLA-4 antibody (including Ipilimumab or any other antibody specifically targeting T-cell co-stimulation or checkpoint pathways).

5. History of severe hypersensitive reactions to other monoclonal antibodies.

6. History of allergy or intolerance to study drug components.

7. Substance abuse, medical, psychological or social conditions that may interfere with the patient's participation in the study or evaluation of the study results.

8. History or concurrent condition of interstitial lung disease of any grade or severely impaired pulmonary function.

9. Uncontrolled intercurrent illness, including ongoing or active systemic infection, symptomatic

congestive heart failure, unstable angina pectoris, cardiac arrhythmia (excluding insignificant sinus bradycardia and sinus tachycardia) or psychiatric illness/social situations and any other illness that would limit compliance with study requirements and jeopardize the safety of the patient.

10. History of human immunodeficiency virus (HIV) infection or acquired immunodeficiency syndrome (AIDS).

11. Pregnant or breast-feeding. Women of childbearing potential must have a pregnancy test performed within 7 days before the enrollment, and a negative result must be documented.

12. Previous or concurrent cancer within 3 years prior to treatment start EXCEPT for curatively treated cervical cancer in situ, non-melanoma skin cancer, superficial bladder tumors [Ta (non-invasive tumor), Tis (carcinoma in situ) and T1 (tumor invades lamina propria)].

13. Vaccination within 30 days of study enrollment.

14. Active bleeding or known hemorrhagic tendency.

15. Subjects with unhealed surgical wounds for more than 30 days.

16. Being participating any other trials or withdraw within 4 weeks.

3.3 Concomitant treatments

Appropriate auxiliary drug, such as liver-protective, heart-protective and stomach-protective, are permitted to perform concomitant with investigational drugs. When adverse events occurred, medical interventions will be permitted.

Concurrent chemotherapy, radiotherapy, and other regional therapies are not permitted in this study.

3.4 Discontinuation of investigational product

Subjects must be discontinued from investigational product in the following situations:

a. Subject decision. The subject is at any time free to discontinue treatment, without prejudice to further treatment.
b. Adverse Event which may be risk to patients as judged by the investigator.
c. Pregnancy.
d. Severe non- compliance with the study protocol as judged by the investigator.
e. Patient incorrectly initiated on investigational product.
f. Objective disease progression or subjects is no longer receiving clinical benefit.
g. Subjects loss to follow-up.

Subjects that decides to discontinue investigational product will always be asked about the reasons and the presence of any adverse events. If possible, they will be seen and assessed by the investigator. Besides, any subject who discontinues study treatment for reasons other than objective disease progression should have clinical assessment performed as scheduled in the protocol until disease progression or death occurs, unless consent is withdrawn.

4 TREATMENTS

All protocol-specified investigational and non-investigational products are considered study drug.

4.1 Investigational Products and Non-investigational Products

The investigational products should be stored in a secure area according to storage requirements. In this protocol, investigational products are nivolumab, gemcitabine and cisplatin.

Non-investigational products used in this study was support or escape medication for preventative,

diagnostic, or therapeutic reasons.

4.2 Handling and Dispensing

The study drugs should be stored in accordance with the environmental conditions (temperature, light, and humidity) or storage instructions on the package insert.

4.3 Preparation and Administration

Preparation:

Nivolumab is stored at refrigerated temperatures (2-8°C), gemcitabine and cisplatin are stored at normal temperature. It ensures that the solution is clear, colorless and free from particulate matter on visual inspection. If multiple vials are needed for a subject, it is important to use a separate sterile syringe and needle for each vial to prevent problems such as dulling of needle tip, stopper coring, repeated friction of plunger against syringe barrel wall, etc. Do not enter into each vial more than once. Any partial vials should be safely discarded per the sites standard operating procedures (SOPs) and should not be reused.

Administration:

Nivolumab is to be administered as a 60-minute IV infusion, using a volumetric pump with a 0.2/0.22 micron in-line filter at the protocol-specified doses. It is not to be administered as an IV push or bolus injection. At the end of the infusion, flush the line with 0.9% normal saline. Gemcitabine is to be administered as a 30-minute IV infusion. Cisplatin is to be administered via IV infusion without time limitation.

4.4 Dose reduction or Discontinuation of study drugs

Study drugs should be permanently discontinued for the following:

a. Any Grade 2 drug-related uveitis, eye pain or blurred vision that does not respond to topical therapy and does not improve to Grade 1 severity or require systemic treatment.

b. Any Grade 3 or higher drug-related bronchospasm, hypersensitivity reaction, or infusion related reaction regardless of duration requires discontinuation.

c. Grade 3 or higher drug-related thrombocytopenia > 7 days associated with bleeding requires discontinuation.

d. Any of the following drug-related liver function test (LFT) abnormalities that meets the following criteria require discontinuation:

- AST or ALT > 5 - 10×ULN for > 2 weeks
- AST or ALT > 10×ULN
- Total bilirubin > 5×ULN
- Concurrent AST or ALT > 3×ULN and total bilirubin > 2×ULN

e. Any Grade 4 drug-related adverse event or laboratory abnormality, except for the following events which do not require discontinuation:

- Grade 4 neutropenia ≤7 days
- Grade 4 lymphopenia or leukopenia
- Isolated Grade 4 electrolyte imbalances/abnormalities that are not associated with clinical sequelae and are corrected with supplementation/appropriate management within 72 hours of their onset.

- Any dosing interruption lasting > 6 weeks with the following exceptions:

Feng K, *et al. J Immunother Cancer* 2020; 8:e000367. doi: 10.1136/jitc-2019-000367

√ Dosing interruptions to allow for prolonged steroid tapers to manage drug related adverse events are allowed.

√ Dosing interruptions > 6 weeks that occur for non-drug-related reasons may be allowed.

The following events which do not require discontinuation require 20%-25% dose reduction for chemotherapeutic drugs in the next cycle treatment:

- Grade 3 or higher drug-related neutropenia
- Grade 3 or higher drug-related thrombocytopenia without association of bleeding.
- Grade 3 or higher drug-related nausea or vomiting.

5 ADVERSE EVENTS AND CLINICAL ADMINISTRATION

5.1 Definition of adverse events

An adverse event is the development of an undesirable medical condition or the deterioration of a pre-existing medical condition following or during exposure to a pharmaceutical product, whether or not considered causally related to the product. An AE not only include an undesirable medical condition, but also involving run-in or washout periods, even if no study treatment has been administered in clinical studies. The term of AE is used to include both serious and non-serious AEs.

5.2 Definitions of serious AE

A SAE is an AE occurring during any study phase that meet the following criteria:

a) Results in death.

b) Is immediately life-threatening, which means the patients lies in risk of death from the AEs

or it is suspected that use or continued use of the drug would result in patients' death.

c) Requires in-patient hospitalization or prolongation of existing hospital.

d) Results in persistent or significantly disability/incapacity or disruption of the ability to conduct normal life functions.

e) Is a congenital abnormality or birth defect.

f) Is an important medical event that might need medical intervention to prevent one of the outcomes listed above.

5.3 Recording of AEs

AEs will be collected from time of receiving the products throughout the treatment period and safety follow-up period. The safety follow-up period is defined as 120 days after treatment is discontinued.

After this study over, there might be some patients remaining on study treatment. These patients will continue to collect information about AEs.

Any AEs that are unresolved are followed up by the investigator for as long as medically indicated. If any investigator learns of any SAEs, including death, at any time, and considers there is a reasonable possibility that the events, the results need document.

5.4 Collected information for AEs

The following variables will be collected for each AE:

AEs	AE;

	The data when the AE stared and stopped;<br>Changes in CTCAE grade;<br>The serious grade of the AE;<br>Action taken with regard to investigational product;<br>Outcome;<br>Treatment measures.
SAEs	Date AE met criteria for SAE;<br>The cause of serious AE;<br>Date of hospitalization;<br>Date of discharge;<br>Causality assessment in relation to study procedures;<br>Description of AE.

All events with an assigned CTCAE grading use the grading scales found in the current National Cancer Institute CTCAE version 5.0. For those events without assigned CTCAE grades, the recommendation in the CTCAE criteria that converts mild, moderate and severe events into CTCAE grades should be used.

5.5 AEs based on signs and symptoms

All AEs that were reported by the patient or care provider in response to the open question from the study personnel, or that were revealed by observation will be collected and recorded in the CRF. When collecting AEs, the recording of diagnoses is preferred to record a list of signs and symptoms. However, if a diagnosis is known and there are other signs or symptoms that are not generally part of

the diagnosis, the diagnosis and each sign or symptom will be recorded separately.

5.6 AEs based on examination and tests

The results from protocol mandated laboratory tests and vital signs will be summarized in the clinical study report. Deterioration as compared to baseline in protocol-mandated parameters should therefore only be reported as AEs if they fulfil any of the SAE criteria or are the reason for discontinuation of treatment with the products unless due to progression of disease under study.

If deterioration in d laboratory value, vital sign, ECG or other safety assessment is associated with clinical signs and symptoms, the sign or symptom will be reported as an AE and the associated laboratory result or other finding will be considered as additional information.

Deterioration of a laboratory value, which is clearly due to disease progression, should not be reported as an AE or SAE.

5.7 Disease progression

Disease progression, including the increase in the severity of the disease and/or increases in the symptoms of the disease, is a worsening of a patient's condition attributable to the disease for which the investigational product is being studied. The development of new, or progression of existing masses should be considered as disease progression and not an AE. Events, which are unequivocally due to disease progression, should not be reported as an AE during the treatment.

5.8 The report of deaths

All deaths during the study or within the follow-up period should be reported. If death is directly

due to disease progression, it should be communicated to the study monitor at the next monitoring visit and should be documented in the CRF module, but should not be reported as a SAE in the study; When death is not clearly due to disease progression of the disease under study the AE causing the death should be reported to the study monitor as an SAE within 24 hours. The report should assign the primary cause of death with any contributory causes.

5.9 Management of the treatment related toxicities

If subjects experience unacceptable toxicity, the subject should be withdrawn from the study and observed until resolution of the toxicity.

If subjects show a CTCAE grade≥3 toxicity during the study therapy, and the investigator consider the AE of concern to be specifically associated with investigational drugs, the treatment will be suspended, and supportive therapy administered as required in accordance with guidelines. If the toxicity resolves within 3 weeks, treatment with investigational drugs may be restarted according to the dose reduction principles.

6 STATISTICAL ANALYSES OF TREATMENT

6.1 Statistical considerations

A comprehensive statistical analysis plan will be prepared prior to first subject enrolled. The data cut-off will be taken place at least 6 months after the last subject enrolled, to ensure that the objective of PFS at 6 months will be analyzed. Meanwhile, the primary endpoint, other secondary endpoints, and exploratory endpoints will be analyzed.

Feng K, *et al. J Immunother Cancer* 2020; 8:e000367. doi: 10.1136/jitc-2019-000367

6.2 Sample size estimate

Currently, the reported ORR of anti-PD-1 antibody in advanced BTCs was 17% and ORR of gemcitabine-based chemotherapy was approximately 20%, and we estimated that if we assume a ORR rate of 55% with anti-PD-1 antibody plus gemcitabine and cisplatin combination. At least 25 patients would need to be enrolled with a two-sided significant level of 0.05 and 90% power.

6.3 Outcome measures for analysis

Response Evaluation Criteria in Solid Tumors (RECIST version 1.1)

From the investigators review of the imagining scans, RECIST version 1.1 will be used to evaluate tumor response data. It will also be used to determine the endpoints ORR, DCR, PFS and OS from the overall visit response and scan dates.

At each evaluation, subjects will be assigned an RECIST version 1.1 response of CR, PR, SD or PD depending on the status of their disease compared with baseline assessments. If a subject has a tumor assessment which cannot be evaluated, then the subject will be assigned a visit response of not evaluable (NE) (unless there is evidence of progression in which case the response will be assigned as PD).

Overall Response Rate (ORR)：

ORR is defined as the number (%) of patients with measurable disease with at least one response evaluation of CR or PR that is confirmed at least 6 weeks later. Data obtained up until progression, or last evaluable assessment in the absence of progression, will be included in the assessment of ORR. However, any CR or PR which occurred after a further anticancer therapy will not be included in numerator of the ORR calculation.

Disease control rate (DCR)：

Disease control rate is defined as the percentage of subjects who have a CR, PR or SD.

Progression Free Survival (PFS)：

PFS is defined as the time from the first dose until the date of objective disease progression or death (by any cause in the absence of progression) regardless of whether the patient withdraws from study therapy or receives another anticancer treatment prior to progression. Subjects who have not progressed or died at the time of analysis will be censored at the time of the latest date of assessment from their last evaluable lymphoma baseline. The PFS time will always be derived based on scan/assessment dates not visit dates.

Overall Survival (OS)：

Overall survival is defined as the time from the first dose until death due to any cause. Any patient not known to have died at the time of analysis will be censored based on the last record date on which the patients was known to be alive.

6.4 Safety Assessments

Adverse events will be listed individually by patient. Any AE occurring within 120 days of last dose of study drugs will be included in the AE summaries. For change from baseline summaries for vital signs, laboratory data and physical examination, the baseline value will be the latest result obtained prior to the start of the first dose of study drugs. All enrolled patients will be included for safety assessments.

6.5 Tumor PD-L1/PD-L2 multiplex immunofluorescence assessment

Feng K, *et al. J Immunother Cancer* 2020; 8:e000367. doi: 10.1136/jitc-2019-000367

Tumor samples at baseline and in the course of treatment will be obtained by excisional biopsy or with a core needle, followed by formalin fixation and paraffin embedding. Tumor PD-L1/PD-L2 expression will be detected by multiplex immunofluorescence tissue staining as followed. Slides are deparaffinised in xylene and rehydrated in a series of graded alcohols. Antigen retrievals are performed in citrate buffer (pH6) with a microwave (Sharp, R-331ZX) for 20 min at 95°C followed by a 20 min cool down at room temperature. After the quenching of endogenous peroxidase in 3% H2O2, slides are incubated with blocking reagent (ZSGB-BIO, ZLI-9022) for 30 min at room temperature. Antigens are then successively detected using the Opal protocol. Briefly, each primary antibody is incubated for 2 hr in a humidified chamber at 37°C, followed by detection using the horseradish peroxidase (HRP)-conjugated secondary antibody (GBI Labs, Polink-1 HRP polymer detection kit) and TSA-fluorophores (PerkinElmer, Opal 7-color IHC Kit, NEL797001KT, 1:100, 20-60 sec), after which the primary and secondary antibodies are thoroughly eliminated by heating the slides in citrate buffer (pH 6.0) for 10 min at 95°C using microwave. In a serial fashion, each antigen is labeled by distinct fluorophores. Nuclei are subsequently visualized with DAPI (1:2000), and the slides are coverslipped using ProLong Gold Antifade Mountant (ThermoFisher, P36934). Using this Opal protocol, PD-L2 (CST, 82723, 1:100, Opal 570), PD-L1 (WuXi AppTec, AW5698, Opal 520), CD30 (Abcam, ab187367, 1:200, Opal 690), CD3 (Abcam, ab16669, 1:200, Opal 620) are sequentially detected within sample sections. For each patient sample, 5-10 fields of view are acquired at 20 × resolution as multispectral images by using the Vectra Polaris (PerkinElmer), depending on tumor size. After image capture, each field of view is spectrally unmixed, cell segmentation and cell quantitation using the inform Advanced Image Analysis Software (PerkinElmer) version 2.4.

6.6 Peripheral blood T cell assessment

Blood samples will be collected for peripheral T cell assessment prior to each cycle of study treatment. The peripheral blood is collected in sodium heparin anticoagulant vacutainer tubes. Briefly, 100 μl of the anticoagulant peripheral blood is incubated with antibodies specific to cell-surface antigens expressed on T lymphocytes. After red blood cell lysis and washing, the cells are detected on a BD FACSCalibur flow cytometer (BD Biosciences). The following antibodies are used to detect surface marker expression and obtained from BD Biosciences: anti-CD3-PerCP (347344), anti-HLA-DR (559866) and isotype-matched antibodies. For the intracellular cytokine expression, blood cells are stimulated with T cell stimulation cocktail (including PMA, Ionomycin and transport inhibitors, eBioscience, 00-4975-93) for 4 h of incubation, and cells were stained with anti-CD3 and permeabilized before the addition of anti-IFN-γ (554700). Stained cells are detected by flow cytometry using FACSCalibur flow cytometer (BD Biosciences) to collect a minimum of 10,000 CD3+ lymphocytes.

6.7 Methods for statistical analyses

Appropriate descriptive statistics will be used for all variables. Continuous variables will be summarized by the number of observations, mean, standard deviation, median, minimum, and maximum. Categorical variable will be summarized by frequency counts and percentages for each category. Unless otherwise stated, percentages will be calculated out of the full analysis set.

ORR will be presented together with two-sided 95% exact confidence interval (CI), which is calculated using the Clopper-Pearson method. Summaries of the number and percentage of

Feng K, *et al. J Immunother Cancer* 2020; 8:e000367. doi: 10.1136/jitc-2019-000367

patients with best response in each of the follow categories will be summarized: complete remission (CR), partial response (PR), stable disease (SD), progressive disease (PD) and non-evaluable (NE).

The best absolute change in target lesion tumor size from baseline and percentage change in target lesion tumor size from baseline will be summarized using descriptive statistics and presented at each time point.

PFS will be displayed using a Kaplan-Meier plot. The number events, median, and the proportion of patients without an event at 6 months summarized.

OS will be displayed using a Kaplan-Meier plot. The number events, median, and the proportion of patients without an event at 12, 18 months will be summarized. Summaries of the number and percentage of patients who died, still in survival follow-up, lost to follow-up or withdraw from study will be presented.

7 LIST OF ABBREVIATIONS

AE	adverse event
ALT	alanine aminotransferase
ANC	absolute neutrophil count
AST	aspartate aminotransferase
BOR	best overall response
BTCs	biliary tract cancers
BUN	blood urea nitrogen
CI	confidence interval
cm	centimeter
CNS	central nervous system
CR	complete remission
CRF	Case Report Form
CT	Computed tomography
CTCAE	Common Terminology Criteria for Adverse Events
CTAL-4	Cytotoxic T lymphocyte-associated antigen 4
ECG	electrocardiogram
ECOG	Eastern Cooperative Oncology Group
ELISA	enzyme-linked immunosorbent assay
ESR	Expedited Safety Report
FDA	Food and Drug Administration
GCP	Good Clinical Practice

HBV	hepatitis B virus
HCV	hepatitis C virus
HRP	horseradish peroxidase
ICF	Informed Consent
ICH	International Conference on Harmonisation
IHC	immunohistochemistry
IRB	Institutional Review Board
IV	Intravenous
Kg	kilogram
MRI	Magnetic Resonance Imaging
mAbs	Monoclonal Antibodys
mg/kg	Milligram per kilogram
NE	non-evaluable
ORR	Objective Response Rate
OS	Overall survival
PBMCs	peripheral blood mononuclear cells
PD	progressive disease
PD-1	Programmed death 1
PD-L1	Programmed death ligand 1
PD-L2	Programmed death ligand 2
PET	Positron emission tomography

Feng K, *et al. J Immunother Cancer* 2020; 8:e000367. doi: 10.1136/jitc-2019-000367

PFS	progression free survival
PR	partial remission
PS	Performance Status
RECIST	Response Evaluation Criteria in Solid Tumors
RT-PCR	reverse transcription polymerase chain reaction
RNA	Ribonucleic acid
SAE	serious adverse event
SD	Stable Disease
TCR	T cell receptor
WBC	white blood cell
WHO	World Health Organization

8 REFERENCE

1. Bray F, Ferlay J, Soerjomataram I, Siegel RL, Torre LA, Jemal A. Global cancer statistics 2018: GLOBOCAN estimates of incidence and mortality worldwide for 36 cancers in 185 countries. CA Cancer J Clin. 2018;68(6):394-424.

2. GBD 2016 Disease and Injury Incidence and Prevalence Collaborators. Global, regional, and national incidence, prevalence, and years lived with disability for 328 diseases and injuries for 195 countries, 1990-2016: a systematic analysis for the Global Burden of Disease Study 2016. Lancet. 2017; 390(10100):1211-1259.

3. Razumilava N, Gores GJ. Classification, Diagnosis, and Management of Cholangiocarcinoma. Clin Gastroenterol Hepatol. 2013; 11(1): 13–e4.

4. Valle J, Wasan H, Palmer DH, Cunningham D, Anthoney A, Maraveyas A, et al. Cisplatin plus Gemcitabine versus Gemcitabine for Biliary Tract Cancer. N Engl J Med. 2010;362(14):1273-1281.

5. Malka D, Cervera P, Foulon S, Trarbach T, de la Fouchardière C, Boucher E, et al. Gemcitabine and oxaliplatin with or without cetuximab in advanced biliary-tract cancer (BINGO): a randomised, open-label, non-comparative phase 2 trial. Lancet Oncol. 2014;15(8):819-828.

6. Park K, Kim KP, Park S, Chang HM. Comparison of gemcitabine plus cisplatin versus capecitabine plus cisplatin as first-line chemotherapy for advanced biliary tract cancer. Asia Pac J Clin Oncol. 2017;13(1):13-20.

7. Rizvi S, Gores GJ. Emerging molecular therapeutic targets for cholangiocarcinoma. J Hepatol. 2017;67(3):632-644.

8. Heymach J, Krilov L, Alberg A, Baxter N, Chang SM, Corcoran R, et al. Clinical Cancer Advances 2018: Annual Report on Progress Against Cancer from the American Society of Clinical Oncology. J Clin Oncol. 2018;36(10):1020-1044.

9. Martin-Liberal J, Ochoa de Olza M, Hierro C, Gros A, Rodon J, Tabernero J. The expanding role of immunotherapy. Cancer Treat Rev. 2017; 54:74-86.

10. Gani F, Nagarajan N, Kim Y, Zhu Q, Luan L, Bhaijjee F, et al. Program Death 1 Immune Checkpoint and Tumor Microenvironment: Implications for Patients With Intrahepatic Cholangiocarcinoma. Ann Surg Oncol. 2016;23(8):2610-2617.

11. Sabbatino F, Villani V, Yearley JH, Deshpande V, Cai L, Konstantinidis IT, et al. PD-L1 and HLA Class I Antigen Expression and Clinical Course of the Disease in Intrahepatic Cholangiocarcinoma. Clin Cancer Res. 2016;22(2):470-478.

12. Nakamura H, Arai Y, Totoki Y, Shirota T, Elzawahry A, Kato M, et al. Genomic spectra of biliary tract cancer. Nat Genet. 2015;47(9):1003-1010.

13.Bang YJ, Doi T, De Braud F, et al. Safety and efficacy of pembrolizumab (MK-3475) in patients (pts) with advanced biliary tract cancer: interim results of KEYNOTE-028. Eur J Cancer 2015;51(3): s112.

14. Le DT, Durham JN, Smith KN, Wang H, Bartlett BR, Aulakh LK, et al. Mismatch repair deficiency predicts response of solid tumors to PD-1 blockade. Science. 2017;357(6349):409-413.

15. Silva VW, Askan G, Daniel TD, Lowery M, Klimstra DS, Abou-Alfa GK, et al. Biliary carcinomas: pathology and the role of DNA mismatch repair deficiency. Chin Clin Oncol. 2016;5(5):62.

16. Wumkes ML, van der Velden AM, Los M, Leys MB, Beeker A, Nijziel MR, et al. Serum antibody response to influenza virus vaccination during chemotherapy treatment in adult patients with solid tumors. Vaccine. 2013;31(52):6177-6184.

17. Garg AD, Vara Perez M, Schaaf M, Agostinis P, Zitvogel L, Kroemer G, et al.Trial watch: Dendritic cell-based anticancer immunotherapy. Oncoimmunology. 2017;6(7): e1328341.

18. Galluzzi L, Buqué A, Kepp O, Zitvogel L, Kroemer G. Immunological Effects of Conventional Chemotherapy and Targeted Anticancer Agents. Cancer Cell. 2015;28(6):690-714.

19. Brahmer JR, Drake CG, Wollner I, Powderly JD, Picus J, Sharfman WH, et al. Phase I study of single-agent anti-programmed death-1 (MDX-1106) in refractory solid tumors: safety, clinical activity, pharmacodynamics, and immunologic correlates. J Clin Oncol. 2010;28(19):3167-3175.

20. Di Caro G, Cortese N, Castino GF, Grizzi F, Gavazzi F, Ridolfi C, et al. Dual prognostic significance of tumor-associated macrophages in human pancreatic adenocarcinoma treated or untreated with chemotherapy. Gut. 2016;65(10):1710-1720.

21. Liu WM, Fowler DW, Smith P, Dalgleish AG. Pre-treatment with chemotherapy can enhance the antigenicity and immunogenicity of tumors by promoting adaptive immune responses. Br J Cancer. 2010;102(1):115-123.

22. Lesterhuis WJ, Punt CJ, Hato SV, Eleveld-Trancikova D, Jansen BJ, Nierkens S, et al. Platinum-based drugs disrupt STAT6-mediated suppression of immune

responses against cancer in humans and mice. J Clin Invest. 2011;121(8):3100-3108.

23. Chen C, Chen Z, Chen D, Zhang B, Wang Z, Le H. Suppressive effects of gemcitabine plus cisplatin chemotherapy on regulatory T cells in non-small cell lung cancer. J Int Med Res. 2015;43(2):180-187.

APPENDIX 1 Response Evaluation Criteria in Solid Tumors version 1.1

1.1 Time point response: patients with target (+/–non-target) disease

Target lesions	Non-target lesions	New lesions	Overall response
CR	CR	No	CR
CR	Non-CR/non-PD	No	PR
CR	Not evaluated	No	PR
PR	Non-PD or not all evaluated	No	PR
SD	Non-PD or not all evaluated	No	SD
Not all evaluated	Non-PD	No	NE
PD	Any	Yes or No	PD
Any	PD	Yes or No	PD
Any	Any	Yes	PD
CR = complete response, PR = partial response, SD = stable disease, PD = progressive disease, and NE = inevaluable.			

1.2 Response criteria

1.2.1 Evaluation of target lesions

Complete Response (CR): Disappearance of all target lesions. Any pathological lymph nodes (whether target or non-target) must have reduction in short axis to <10 mm.

Partial Response (PR): At least a 30% decrease in the sum of diameters of target lesions, taking as reference the baseline sum diameters.

Progressive Disease (PD): At least a 20% increase in the sum of diameters of target lesions, taking as reference the smallest sum on study (this includes the baseline sum if that is the smallest on study). In addition to the relative increase of 20%, the

sum must also demonstrate an absolute increase of at least 5 mm. (Note: the appearance of one or more new lesions is also considered progression).

Stable Disease (SD): Neither sufficient shrinkage to qualify for PR nor sufficient increase to qualify for PD, taking as reference the smallest sum diameters while on study.

1.2.2 Evaluation of non-target lesions

Complete Response (CR): Disappearance of all non-target lesions and normalization of tumor marker level. All lymph nodes must be non-pathological in size (<10mm short axis).

Non-CR/Non-PD: Persistence of one or more non-target lesion (s) and/or maintenance of tumor marker level above the normal limits.

Progressive Disease (PD): Unequivocal progression (see comments below) of existing non-target lesions. (Note: the appearance of one or more new lesions is also considered progression).

Feng K, *et al. J Immunother Cancer* 2020; 8:e000367. doi: 10.1136/jitc-2019-000367

APPENDIX 2 COMMON TERMINOLOGY CRITERIA FOR ADVERSE EVENTS V5.0 (CTCAE)

The descriptions and grading scales found in the revised NCI Common Terminology Criteria for Adverse Events (CTCAE) version 5.0 will be utilized for adverse event reporting(http://ctep.cancer.gov/protocolDevelopment/electronic_applications/ctc.htm).

APPENDIX 3 ECOG PERFORMANCE STATUS SCALE

Grade	Description
0	Normal activity. Fully active, able to carry on all pre-disease performance without restriction.
1	Symptoms, but ambulatory. Restricted in physically strenuous activity, but ambulatory and able to carry out work of a light or sedentary nature (e.g., light housework, office work).
2	In bed <50% of the time. Ambulatory and capable of all self-care, but unable to carry out any work activities. Up and about more than 50% of waking hours.
3	In bed >50% of the time. Capable of only limited self-care, confined to bed or chair more than 50% of waking hours.
4	100% bedridden. Completely disabled. Cannot carry on any self-care. Totally confined to bed or chair.
5	Dead.

Feng K, *et al. J Immunother Cancer* 2020; 8:e000367. doi: 10.1136/jitc-2019-000367

APPENDIX 4 NEW YORK HEART ASSOCIATION (NYHA) CLASSIFICATION

Class I: patients with no limitation of activities; they suffer no symptoms from ordinary activities.

Class II: patients with slight, mild limitation of activity; they are comfortable with rest or with mild exertion.

Class III: patients with marked limitation of activity; they are comfortable only at rest.

Class IV: patients who should be at complete rest, confined to bed or chair; any physical activity brings on discomfort and symptoms occur at rest.

Feng K, *et al. J Immunother Cancer* 2020; 8:e000367. doi: 10.1136/jitc-2019-000367

TABLE OF CONTENTS

TITLE PAGE……………………………………………………………4

SYNOPSIS………………………………………………………………6

1 INTRODUCTION AND STUDY RATIONALE…………………..12

1.1 Biliary tract cancers…………………………………………………12

1.2 Background and rationale for conducting this study………………..13

1.3 Rationale for study design…………………………………………..15

1.4 Research Hypothesis………………………………………………..16

1.5 Objectives…………………………………………… …………..16

1.6 Overall Risk/Benefit Assessment…………………………………...17

2 ETHICAL CONSIDERATIONS…………………………………...17

2.1 Ethical conduct of the study……………………………………......17

2.2 Institutional Review Board/Independent Ethics Committee………..18

2.3 Subjects data protection……………………………………………..19

2.4 Informed Consent…………………………………………………......19

2.5 Change to the protocol and informed consent form………………...21

2.6 Audits and inspections………………………………………………21

3 INVESTIGATIONAL PLAN……………………………………..22

3.1 Study design and duration…………………………………………..22

3.2 Study population…………………………………………………….24

3.3 Concomitant treatments……………………………………………..27

3.4 Discontinuation of investigational product…………………………27

4 TREATMENTS……………………………………………………..… 28

4.1 Investigational Products and Non-investigational Products………...28

4.2 Handling and Dispensing…………………………………………...28

4.3 Preparation and Administration…………………………………….28

4.4 Dose reduction or Discontinuation of study drugs…………………29

5 ADVERSE EVENTS AND CLINICAL ADMINISTRATION…...31

5.1 Definition of adverse events……………………………………......31

5.2 Definitions of serious AE…………………………………………...31

5.3 Recording of AEs………………………………………..…….32

5.4 Collected information for AEs……………………………………32

5.5 AEs based on signs and symptoms………………………………….33

5.6 AEs based on examination and tests………………………………...34

5.7 Disease progression…………………………………………………34

5.8 The report of deaths…………………………………………………35

5.9 Management of the treatment related toxicities…………………….35

6 STATISTICAL ANALYSES OF TREATMENT……………….....36

6.1 Statistical considerations…………………………………………....36

6.2 Sample size estimate………………………………………………...36

6.3Outcome measures for analysis……………………………………...36

6.4 Safety Assessments……………………………………………….....38

6.5 Tumor PD-L1 assessment………...38

6.6 Peripheral blood T cell assessment………………………………….39

6.7 Methods for statistical analyses……………………………………39

7 LIST OF ABBREVIATIONS……………………………………....41

8 REFERENCES……………………………………………………....44

APPENDIX 1 Response Evaluation Criteria in Solid Tumors……...48

APPENDIX 2 COMMON TERMINOLOGY CRITERIA FOR ADVERSE EVENTS V5.0 (CTCAE)…………………………………50

APPENDIX 3 ECOG Performance Status Scale…………………….51

APPENDIX 4 New York Heart Association (NYHA) Classification..52

Chinese PLA General Hospital

Title Page

Efficacy analysis of nivolumab in combination with gemcitabine and cisplatin in patients with unresectable or metastatic biliary tract cancers

Principal Investigator Weidong Han MD PhD
Bio-therapeutic Department
Molecular & Immunological Department
Chinese PLA General Hospital
Beijing 100853 China
Co-Investigator Kaichao Feng MD
Qian Mei PhD
Yang Liu MD
Qingming Yang MD
Liang Dong BA
Jiejie Liu BA
Xiang Li BA
Regulatory Sponsor Weidong Han MD PhD
Bio-therapeutic Department
Molecular & Immunological Department
Chinese PLA General Hospital
Beijing 100853 China
Funding Sponsor National Natural Science Foundation of China
National Key Research and Development Program of China
Study Product Nivolumab; Gemcitabine; Cisplatin
Protocol Version 2 May 15, 2017
CONFIDENTIAL
This document is confidential and the property of Chinese PLA General Hospital. No part of it may be transmitted, reproduced, published, or used by other persons without prior written authorization from the study sponsor.

STUDY TEAM

Clinical Trial Site-Chinese PLA General Hospital

Principal Investigator
Weidong Han MD PhD

Clinical Team
Kaichao Feng MD
Yang Liu MD
Qingming Yang MD

Laboratory Team
Qian Mei PhD
Liang Dong BA
Jiejie Liu BA
Xiang Li BA

Statistical Support
Qian Mei PhD

Feng K, et al. J Immunother Cancer 2020; 8:e000367. doi: 10.1136/jitc-2019-000367

SYNOPSIS

Protocol Title: Efficacy analysis of nivolumab in combination with gemcitabine and cisplatin in patients with unresectable or metastatic biliary tract cancers

Investigational Product(s), Dose and Mode of Administration, Duration of Treatment with Investigational Product(s): Gemcitabine dosed at 1000 mg/m^2 on day 1 and day 5, cisplatin dosed at 75 mg/m^2 on day 1, and nivolumab dosed at 3 mg/kg on day 3 will be given intravenously every 3 weeks as a cycle for a maximum of 6 cycles. Patients who respond to the combination treatment or obtain stable disease will switch to maintenance therapy with nivolumab and gemcitabine every six to eight weeks until disease progression, intolerable toxicity, death, withdrawal of consent or any other reasons.

Study Phase: II

Research Hypothesis: The combination of nivolumab with standard gemcitabine and cisplatin chemotherapy could improve the overall response rate and survival for patients with advanced BTCs without aggravated toxicities.

Study Objectives:

Primary Objective: To evaluate the overall response rate (ORR) of nivolumab in combination with gemcitabine and cisplatin for advanced unresectable or metastatic BTCs.

Secondary Objectives: To evaluate safety and other efficacy parameters, including the disease control rate (DCR), progression free survival (PFS), PFS at 6 months, overall survival (OS) and OS at 12 months.

Exploratory Objectives: To evaluate pathological, immunological or clinical predictive factors for response and prognosis.

Study Design: Subjects will receive study drug as detailed in Figure 1

Figure 1:

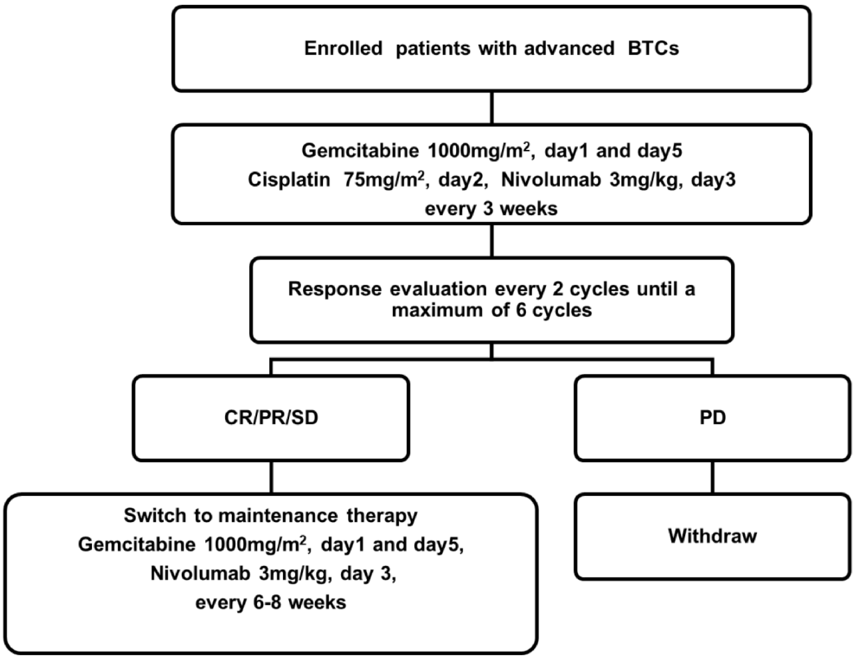

Study Population:

Eligible subjects should meet all criteria of inclusion and exclusion, details as follows：

Inclusion Criteria:

1. Age from 18 to 75 years with estimated life expectancy >3 months.

2. Histopathological confirmed advanced unresectable or metastatic BTCs and had at least one measurable disease (≥1cm).

3. Subjects should provide fresh tumor tissue samples or formalin-fixed paraffin embedded tumor archival samples within 3 months and willing to accept site-matched tumor re-biopsy in the process of the study.

4. Prior radiotherapy, chemotherapy, or other local ablative therapies must have been completed ≥ 4 weeks prior to enrollment and patient must has recovered to <= grade 1 toxicity.

5. ECOG (Eastern Cooperative Oncology Group) performance status of 0-2.

6. Adequate organ and marrow function obtained ≤ 2 weeks before enrollment as defined below:

Leukocytes greater than or equal to 3.0 x 10^9/L.

Absolute neutrophil count greater than or equal to 1.0 x 10^9/L.

Platelets greater than or equal to 100 x 10^9/L.

Hemoglobin greater than or equal to 90 g/L.

Total bilirubin less than or equal to 2 x ULN.

Serum albumin should be no less than 25g/L.

ALT or AST less than 2 x ULN.

Measured creatinine clearance ≥60 mL per min.

7. Ability to understand and willingness to sign a written informed consent document.

8. Women of child-bearing potential must agree to use adequate contraception (hormonal or barrier method of birth control; abstinence) prior to study entry, and up to 120 days after the last dose of the drug.

Exclusion Criteria:

1. Active, known or suspected autoimmune diseases.

2. Known brain metastases or active central nervous system (CNS). If patients with CNS metastases were treated with radiotherapy for at least 3 months prior to enrollment and have no central nervous symptoms and are off corticosteroids, they will be eligible but will need a brain MRI prior to enrollment.

3. Subjects are being treated with either corticosteroids (>10 mg daily prednisone equivalent) or other immunosuppressive medications within 14 days of enrollment.

4. Prior therapy with anti-PD-1, anti-PD-L1, or anti-CTLA-4 antibody (including Ipilimumab or any other antibody specifically targeting T-cell co-stimulation or checkpoint pathways).

5. History of severe hypersensitive reactions to other monoclonal antibodies.

6. History of allergy or intolerance to study drug components.

7. Substance abuse, medical, psychological or social conditions that may interfere with the patient's participation in the study or evaluation of the study results.

8. History or concurrent condition of interstitial lung disease of any grade or severely impaired pulmonary function.

9. Uncontrolled intercurrent illness, including ongoing or active systemic infection, symptomatic congestive heart failure, unstable angina pectoris, cardiac arrhythmia (excluding insignificant sinus bradycardia and sinus tachycardia) or psychiatric illness/social situations and any other illness that would limit compliance with study requirements and jeopardize the safety of the patient.

10. History of human immunodeficiency virus (HIV) infection or acquired

Feng K, *et al. J Immunother Cancer* 2020; 8:e000367. doi: 10.1136/jitc-2019-000367

immunodeficiency syndrome (AIDS).

11. Pregnant or breast-feeding. Women of childbearing potential must have a pregnancy test performed within 7 days before enrollment, and a negative result must be documented.

12. Previous or concurrent cancer within 3 years prior to enrollment EXCEPT for curatively treated cervical cancer in situ, non-melanoma skin cancer, superficial bladder tumors [Ta (non-invasive tumor), Tis (carcinoma in situ) and T1 (tumor invades lamina propria)].

13. Vaccination within 30 days of study enrollment.

14. Active bleeding or known hemorrhagic tendency.

15. Subjects with unhealed surgical wounds for more than 30 days.

16. Participating any other trials or withdraw within 4 weeks.

Study Assessments:

Safety assessments: Adverse events will be assessed by National Cancer Institute Common Terminology Criteria for Adverse Events (CTCAE) version 5.0. ALL subjects will be followed up for adverse events up to 120 days after the last dose of study drugs

Efficacy assessments: Computed tomography (CT) scans with contrast or magnetic resonance imaging (MRI) will be used to assess the antitumor efficacy according to Response Evaluation Criteria in Solid Tumors (version 1.1). Positron emission tomography-computed tomography (PET-CT) will be used to confirm the response status when CT or MRI shows a complete response.

Potent biomarkers: Serial blood samples will be collected in the study and site-match tumor re-biopsy will be encouraged when subjects being evaluated as responsive or progressive disease. The peripheral T cell phenotype and activity will be detected by FACS, and tumor cell PD-L1 expression level will be assessed by immunohistochemistry using the Dako 22C3 pharmDx assay. Whole-exome sequencing will be used to assess DNA level from tumor biopsies and matched peripheral-blood mononuclear cell samples.

Endpoints:

Primary endpoints: ORR

Secondary endpoints: DCR, safety, PFS, PFS at 6 months, OS, OS at 12 months.

Exploratory endpoints: pathological, immunological or clinical predictive biomarkers for response and prognosis.

Feng K, *et al. J Immunother Cancer* 2020; 8:e000367. doi: 10.1136/jitc-2019-000367

1 INTRODUCTION AND STUDY RATIONALE

1.1 Biliary tract cancers

Biliary tract cancers (BTCs) represent a diverse group of highly invasive heterogeneous epithelial cancers arising from the biliary tract. Base on their anatomic location, BTCs are classified into gallbladder carcinoma (GBCA), intrahepatic cholangiocarcinoma (iCCA), perihilar cholangiocarcinoma (pCCA) and distal cholangiocarcinoma (dCCA). The incidence of BTCs has increased globally over the past few decades[1], with reported prevalence of 169,000 patients diagnosed with BTCs in 2016[2]. Surgical resection is potentially curative treatment option for early-stage BTCs, however, most patients with BTCs already have locally advanced or metastatic disease at the time of diagnosis. Even in cases of resection, recurrence is seen in > 60% of patients within the first or the second year[3].

For patients with advanced unresectable and/or metastatic BTCs, chemotherapy is still the main systemic therapy. Gemcitabine in combination with cisplatin was reported achieving a 11.7-month median overall survival and thus were recommended as the standard first-line systemic therapy[4]. Other chemotherapeutic regimens such as gemcitabine and oxaliplatin with or without cetuximab[5], capecitabine plus cisplatin[6], were also tested and showed similar efficacy when compared with gemcitabine and cisplatin as first-line chemotherapy for advanced biliary tract cancers. Recently, there are several trials of small molecule kinase inhibitors in advanced biliary tract cancers by targeting FGFR, IDH, MET, Mesothelin, BRCA and other mutated genes, however, the results are disappointing[7].

1.2 Background and rationale for conducting this study

In recent years, immune checkpoint inhibitors, exemplified by antibodies that target programmed cell death-1 (PD-1) and programmed cell death-ligand 1 (PD-L1), have been studied across a variety of tumors and demonstrated robust and durable anti-tumor activity, coupled with low rates of immune-mediated toxicity[8-9]. Preclinical data have suggested an encouraging future for targeting checkpoint pathways in biliary tract tumors. Multiple studies using immunohistochemistry have observed PD-L1 expression in neoplastic cells and inflammatory cell aggregates in cases of ICC. Gani et al reported PD-L1 expression on cells in the tumor front in 72% of samples of resected ICC. Positive PD-L1 expression was significantly associated with 60% reduction in OS compared to PD-L1 negative counterparts[10]. Sabbatino et al reported PD-1 and PD-L1 expression in 100% of resected ICC specimen and evidence of antitumor T-cell mediated immune responses[11]. Transcriptome sequencing and clustering of gene-expression profiles revealed that a subgroup of patients with biliary tract cancers had a high mutational load, resulting in abundant tumor-specific neoantigens and enrichment of immune-related genes[12].

Currently, clinical data with immune checkpoint inhibitors (ICIs) in biliary tract cancers are very limited. Interim safety and efficacy data from the KEYNOTE-028 basket trial reported that a total of 24 patients with PD-L1 positive biliary tract cancers were enrolled in this study. Of the 24 patients, 4 (17.4%) obtained a partial response, 4 had stable disease, and 12 had disease progression[13]. In another basket

trial, PD-1 blockade with pembrolizumab resulted in 100% disease control rate in 4 patients with tumor DNA mismatch repair (MMR)-deficient cholangiocarcinoma (one of the patients had a complete response, and the other three had stable disease)[14].

Although MMR deficiency and/or microsatellite instability (MSI) is associated with higher response rates and durability of responses to immune-checkpoint blockade, MMR deficiency has been reported to occur in 5- 10% of biliary tract cancers[15], limiting the clinical use of immune checkpoint inhibitors in biliary tract cancers. Meanwhile, Interim analysis of the KEYNOTE-028 basket trial revealed that the sensitivity of PD-L1 positive biliary tract cancers to immune checkpoint inhibitors was low. Thus, strategies that could improve the efficacy of immune checkpoint inhibitors are needed.

Clinician oncologists often argue that conventional chemotherapeutics cannot have positive effects on anticancer immune responses. However, recent studies illustrated that routine dose of chemotherapy could allow for the elicitation of immune response by vaccines against the influenza virus[16]. In line with this notion, experimental DC-, DNA- and peptide-based anticancer vaccines can elicit tumor-targeting immune response in patients treated with conventional chemotherapeutics[17]. In return, immunotherapy could neutralize the unwarranted immunosuppressive effects of anticancer drugs and can be harnessed to maximize the immunostimulatory effects of chemotherapy[18].Therefore, chemotherapy have the potential to interact positively with ICI-based immunotherapy.

1.3 Rationale for study design

Nivolumab

Nivolumab, a genetically engineered, fully human, IgG4 immune checkpoint inhibitor antibody manufactured by Bristol-Myers Squibb, contains an engineered hinge region mutation (S228P) designed to prevent exchange of IgG4 molecules. It binds PD-1 on activated immune cells with high affinity (K_D=2.6 nmol/L by Scatchard analysis to polyclonally activated human T cells) to block PD-1 interaction with PD-L1 and PD-L2 ligands, thereby attenuating inhibitory signals and augmenting the host anti-tumor response[19]. Up to now, nivolumab is approved by FDA for a variety of indications, including unresectable or metastatic melanoma, metastatic non-small cell lung cancer, and renal cell carcinoma.

Gemcitabine

Gemcitabine, a nucleoside analog used commonly for the treatment of pancreatic carcinoma, lung squamous carcinoma, and other cancers, can reduce the amount of circulating MDSCs, favors the reprogramming of TAMs toward an immunostimulatory phenotype, and boosts cross-priming[20]. Besides the direct immunostimulatory effects, gemcitabine stimulates the expression of MHC class I molecule expression in cancer cells, thereby increasing their antigenicity[21].

Cisplatin

Cisplatin, a commonly used cytotoxic chemotherapeutic drug, was reported having the potential to augment immune-stimulating activity in a dose-dependent manner by reducing the expression of T cell inhibitory molecule programmed death

receptor-ligand 2 (PD-L2) on both human dendritic cells and tumor cells, resulting in the enhanced antigen-specific proliferation and Th1 cytokine secretion as well as enhanced recognition of tumor cells by T cells[22]. The combination of cisplatin and gemcitabine could significantly reduce the percentage of regulatory T cells in non-small cell lung cancer[23].

1.4 Research Hypothesis

The combination of nivolumab with standard gemcitabine and cisplatin chemotherapy could improve the overall response rate and survival of patients with advanced BTCs without aggravated toxicities.

1.5 Objectives

1.5.1 Primary Objective:

To evaluate the overall response rate (ORR) of nivolumab in combination with gemcitabine and cisplatin for advanced unresectable or metastatic BTCs.

1.5.2 Secondary Objectives:

To evaluate safety and other efficacy parameters, including the disease control rate (DCR), progression free survival (PFS), PFS at 6 months, overall survival (OS) and OS at 12 months.

1.5.3 Exploratory Objectives:

To evaluate pathological, immunological or clinical predictive factors for response and prognosis.

Feng K, *et al. J Immunother Cancer* 2020; 8:e000367. doi: 10.1136/jitc-2019-000367

1.6 Overall Risk/Benefit Assessment

Biliary tract cancers are highly invasive heterogeneous epithelial cancers with the poorest prognosis. Approximately 90% of the patients with biliary tract cancers were diagnosed with advanced unresectable or metastatic status, resulting the median OS less than 12 months. Even in cases of resection, recurrence is seen in > 60% of patients within the first or the second year. However, current systemic strategy for care is gemcitabine-based chemotherapy, which provide limited benefit for patients with advanced biliary tract cancers.

2 ETHICAL CONSIDERATIONS

2.1 Ethical conduct of the study

This study will be performed in accordance with Good Clinical Practice (GCP), as defined by the International Conference on Harmonization (ICH), and in accordance with the ethical principles underlying the policy of Chinese PLA General Hospital on Bioethics and Human Biological Samples.

The study will be conducted in compliance with the protocol. The protocol and any amendments and the subject informed consent will receive the approval/ favorable opinion from Institutional Review Board/Independent Ethics Committee (IRB/IEC) prior to the study.

Personnel involved in this study will be qualified by education, training, and experience to perform their respective tasks.

2.2 Institutional Review Board/Independent Ethics Committee

As Ethics Committee should approve the final study protocol, including the final version of the Informed Consent form and any other written materials to be provided to the subjects. The investigators should also provide the IRB/IEC with a copy of the Investigator Brochure or product labeling information to be provided to subjects and any updates.

The opinion of the Ethics Committee should be given in written form. The investigators should submit the written approval to Chinese PLA General Hospital before enrollment of any subject into the study.

The Ethics Committee should approve all advertising used to recruit subjects for the study. The research team should approve any modification to the Informed Consent form that are needed to meet local requirements.

If required by the local regulation, the protocol should be re-approved by the Ethics Committee annually.

Before enrollment of any subject into the study, the final study protocol, including the final version of the Informed Consent form, should be approved by the national regulatory authority or a notification to the national regulatory authority is done, according to the local regulations.

Each principal investigator is responsible for providing the Ethics Committee with reports of any serious or unexpected adverse drug reactions from any other study conducted with the investigational product. The research team will provide this

Feng K, *et al. J Immunother Cancer* 2020; 8:e000367. doi: 10.1136/jitc-2019-000367

information to the principal investigator so that he/she can meet the reporting requirements.

2.3 Subjects data protection

The Informed Consent Form will incorporate wording that complies with relevant data protection and privacy legislation.

Precautions are taken to preserve confidentiality and prevent data being linked to the identity of the subjects. In exceptional circumstances, however, certain individual might see both the data and the personal identifiers of a subject. Regulatory authorities may require assess to the relevant files, though the subject's medical information and the genetic files would remain physically separate.

2.4 Informed Consent

Investigators must ensure that subjects are clearly and fully informed about the purpose, potential risks, and other critical issues regarding clinical studies in which they volunteer to participate.

In situations where consent cannot be given to subjects, their legally acceptable representatives are clearly and fully informed about the purpose, potential risks, and other critical issues regarding clinical studies in which the subject volunteers to participate.

Investigators will provide an appropriate (ie, Global or Local) sample informed consent form which will include all elements required by ICH, GCP and applicable

Feng K, *et al. J Immunother Cancer* 2020; 8:e000367. doi: 10.1136/jitc-2019-000367

regulatory requirements. The sample informed consent form will adhere to the ethical principles that have their origin in the Declaration of Helsinki.

Investigators must: 1) Provide a copy of the consent form and written information about the study in the language in which the subject is most proficient prior to clinical study participation. The language must be non-technical and easily understood. 2) Allow time necessary for subject or subject's legally acceptable representative to inquire about the details of the study. 3) Obtain an informed consent signed and personally dated by the subject or the subject's legally acceptable representative and by the person who conducted the informed consent discussion. 4) Obtain the IRB/IEC's written approval/favorable opinion of the written informed consent form and any other information to be provided to the subjects, prior to the beginning of the study, and after any revisions are completed for new information. 5) If informed consent is initially given by a subject's legally acceptable representative or legal guardian, and the subject subsequently becomes capable of making and communicating his or her informed consent during the study, consent must additionally be obtained from the subject. 6) Revise the informed consent whenever important new information becomes available that is relevant to the subject's consent. The investigator, or a person designated by the investigator, should fully inform the subject or the subject's legally acceptable representative or legal guardian, of all pertinent aspects of the study and of any new information relevant to the subject's willingness to continue participation in the study. This communication should be documented.

The rights, safety, and well-being of the study subjects are the most important considerations and should prevail over interests of science and society.

2.5 Change to the protocol and informed consent form

If there are any substantial changes to the study protocol, then these changes will be documented in study protocol amendment and where required in a new version of the study protocol.

The amendment should be approved by the relevant Ethics Committee and if applicable, also by the national regulatory authority before implementation. Local requirements are to be followed for revised protocols.

If local regulations require, any administrative change will be communicated to or approved by each Ethics Committee.

2.6 Audits and inspections

Ethics Committee perform audits or inspections at the center, including source data verification. The purpose of an audit or inspection is to systematically and independently examine all study-related activities and documents, to determine whether these activities were conducted, and data were recorded, analyzed, and accurately reported according to the protocol, Good Clinical Practice (GCP), guideline of the International Conference on Harmonisation (ICH), and any applicable regulatory requirements.

Feng K, *et al. J Immunother Cancer* 2020; 8:e000367. doi: 10.1136/jitc-2019-000367

3 INVESTIGATIONAL PLAN

3.1 Study design and duration

This is a Phase II open-label, single-center, single-arm study of Nivolumab, an anti-PD-1 antibody, in combination with gemcitabine and cisplatin for advanced unresectable or metastatic Biliary tract cancers. The primary objective of this study is to evaluate the overall response rate (ORR) of nivolumab in combination with gemcitabine and cisplatin for advanced unresectable or metastatic BTCs. The secondary objectives include evaluating safety and other efficacy parameters, such as the disease control rate (DCR), progression free survival (PFS), PFS at 6 months, overall survival (OS) and OS at 12 months. The exploratory objectives are to evaluate pathological, immunological or clinical predictive factors for response and prognosis.

This study includes sections of Screening, Treatment, and Follow-up. Patients will receive treatment until disease progression, death, unacceptable toxicity, or withdrawal of consent. After receiving a maximum of 6 cycles of combination treatment, responsive and stable patients (CR, PR and durable SD) will switch to maintenance therapy. Assessment of clinical response will be performed every 2 cycles by CT with contrast or MRI. PET-CT will be required when CT with contrast or MRI showed a complete response. The toxicity assessment following administration of the treatment will also be obtained.

Screening:

a. Begins by establishing subject's initial eligibility and signing of the informed consent form;

Subject is enrolled via outpatient service and obtains a subject ID.

b. Tumor tissue (archival or fresh) is suggested to obtain as possible for exploratory study.

c. Baseline disease, treatment history and tumor assessments should be performed within 2 weeks.

d. Subjects are assessed for the study eligibility within the required timeframe.

Treatment:

a. To characterize safety and efficacy of nivolumab in combination of gemcitabine and cisplatin.

b. Treatment should be administrated within 1 week of enrollment.

c. Clinical examinations and biochemistry tests will be collected before the start of each cycle treatment. Adverse events should be documented every cycle.

d. Samples of peripheral blood will be collected every cycle of treatment for T cell function testing.

e. Subjects who are responsive or resistant to the treatment will be suggested to do re-biopsy of target lesions.

f. All study drugs will be administered intravenously in each cycle until disease progression, serious toxicity, withdrawal of consent.

g. Subjects will be evaluated for response by investigators per immune-related response criteria.

Follow-up:

a. Safety follow-up will be performed up to 120 days after the last dose of study

drugs.

b. Subjects will be followed for PFS and OS every 3 month until death.

c. Subjects who discontinue the study for reasons other than disease progression will be required to continue radiographic assessments every 3 months until death or withdrawal of study consent.

3.2 Study population

Subjects must meet the following criteria for the study enrollment.

3.2.1 Inclusion criteria:

1. Age from 18 to 75 years with estimated life expectancy >3 months.

2. Histopathological confirmed advanced unresectable or metastatic BTCs and had at least one measurable disease (⩾1cm).

3. Subjects should provide fresh tumor tissue samples or formalin-fixed paraffin embedded tumor archival samples within 3 months and willing to accept site-matched tumor re-biopsy in the process of the study.

4. Prior radiotherapy, chemotherapy, or other local ablative therapies must have been completed ⩾ 4 weeks prior to enrollment and patient must has recovered to <= grade 1 toxicity.

5. ECOG (Eastern Cooperative Oncology Group) performance status of 0-2.

6. Adequate organ and marrow function obtained ⩽ 2 weeks before enrollment as defined below:

Leukocytes greater than or equal to 3.0 x 10^9/L.

Absolute neutrophil count greater than or equal to 1.0 x 10^9/L.

Platelets greater than or equal to 100 x 10^9/L.

Hemoglobin greater than or equal to 90 g/L.

Total bilirubin less than or equal to 2 x ULN.

Serum albumin should be no less than 25g/L.

ALT or AST less than 2 x ULN.

Measured creatinine clearance ⩾60 mL per min.

7.Ability to understand and willingness to sign a written informed consent document.

8.Women of child-bearing potential must agree to use adequate contraception (hormonal or barrier method of birth control; abstinence) prior to study entry, and up to 120 days after the last dose of the drug.

3.2.2 Exclusion Criteria:

1. Active, known or suspected autoimmune diseases.

2. Known brain metastases or active central nervous system (CNS). If patients with CNS metastases were treated with radiotherapy for at least 3 months prior to enrollment and have no central nervous symptoms and are off corticosteroids, they will be eligible but will need a brain MRI prior to enrollment.

3. Subjects are being treated with either corticosteroids (>10 mg daily prednisone equivalent) or other immunosuppressive medications within 14 days of enrollment.

4. Prior therapy with anti-PD-1, anti-PD-L1, or anti-CTLA-4 antibody (including Ipilimumab or any other antibody specifically targeting T-cell co-stimulation or checkpoint pathways).

5. History of severe hypersensitive reactions to other monoclonal antibodies.

6. History of allergy or intolerance to study drug components.

7. Substance abuse, medical, psychological or social conditions that may interfere with the patient's participation in the study or evaluation of the study results.

8. History or concurrent condition of interstitial lung disease of any grade or severely impaired pulmonary function.

9. Uncontrolled intercurrent illness, including ongoing or active systemic infection, symptomatic congestive heart failure, unstable angina pectoris, cardiac arrhythmia (excluding insignificant sinus bradycardia and sinus tachycardia) or psychiatric illness/social situations and any other illness that would limit compliance with study requirements and jeopardize the safety of the patient.

10. History of human immunodeficiency virus (HIV) infection or acquired immunodeficiency syndrome (AIDS).

11. Pregnant or breast-feeding. Women of childbearing potential must have a pregnancy test performed within 7 days before enrollment, and a negative result must be documented.

12. Previous or concurrent cancer within 3 years prior to enrollment EXCEPT for curatively treated cervical cancer in situ, non-melanoma skin cancer, superficial bladder tumors [Ta (non-invasive tumor), Tis (carcinoma in situ) and T1 (tumor invades lamina propria)].

13. Vaccination within 30 days of study enrollment.

14. Active bleeding or known hemorrhagic tendency.

15. Subjects with unhealed surgical wounds for more than 30 days.

16. Participating any other trials or withdraw within 4 weeks.

3.3 Concomitant treatments

Appropriate auxiliary drug, such as liver-protective, heart-protective and stomach-protective, are

permitted to perform concomitant with investigational drugs. When adverse events occurred, medical interventions will be permitted.

Concurrent chemotherapy, radiotherapy, and other regional therapies are not permitted in this study.

3.4 Discontinuation of investigational product

Subjects must be discontinued from investigational product in the following situations:

a. Subject decision. The subject is at any time free to discontinue treatment, without prejudice to further treatment.

b. Adverse Event which may be risk to patients as judged by the investigator.

c. Pregnancy.

d. Severe non- compliance with the study protocol as judged by the investigator.

e. Patient incorrectly initiated on investigational product.

f. Objective disease progression or subjects is no longer receiving clinical benefit.

g. Subjects loss to follow-up.

Feng K, *et al. J Immunother Cancer* 2020; 8:e000367. doi: 10.1136/jitc-2019-000367

Subjects that decide to discontinue investigational product will always be asked about the reasons and the presence of any adverse events. If possible, they will be seen and assessed by the investigator. Besides, any subject who discontinues study treatment for reasons other than objective disease progression should have clinical assessment performed as scheduled in the protocol until disease progression or death occurs, unless consent is withdrawn.

4 TREATMENTS

All protocol-specified investigational products are considered study drugs.

4.1 Investigational Products and Non-investigational Products

The investigational products should be stored in a secure area according to the storage requirements. In this protocol, investigational products are nivolumab, gemcitabine and cisplatin.

Non-investigational products used in this study are to support or escape medication for preventative, diagnostic, or therapeutic reasons.

4.2 Handling and Dispensing

The study drugs should be stored in accordance with the environmental conditions (temperature, light, and humidity) or storage instructions on the package insert.

4.3 Preparation and Administration

Preparation:

Nivolumab is stored at refrigerated temperatures (2-8°C), gemcitabine and cisplatin are stored at normal temperature. It ensures that the solution is clear, colorless and free from particulate matter on visual inspection. If multiple vials are needed for a subject, it is important to use a separate sterile syringe and needle for each vial to prevent problems such as dulling of needle tip, stopper coring, repeated friction of plunger against syringe barrel wall, etc. Do not enter into each vial more than once. Any partial vials should be safely discarded per the sites standard operating procedures (SOPs) and should not be reused.

Administration:

Nivolumab is to be administered as a 60-minute IV infusion, using a volumetric pump with a 0.2/0.22 micron in-line filter at the protocol-specified doses. It is not to be administered as an IV push or bolus injection. At the end of the infusion, flush the line with 0.9% normal saline. Gemcitabine is to be administered as a 30-minute IV infusion. Cisplatin is to be administered via IV infusion without time limitation.

4.4 Dose reduction or Discontinuation of study drugs

Study drugs should be permanently discontinued for the following:

a. Any Grade 2 drug-related uveitis, eye pain or blurred vision that does not respond to topical therapy and does not improve to Grade 1 severity or require systemic treatment.

b. Any Grade 3 or higher drug-related bronchospasm, hypersensitivity reaction, or

infusion related reaction regardless of duration requires discontinuation.

c. Grade 3 or higher drug-related thrombocytopenia > 7 days associated with bleeding requires discontinuation.

d. Any of the following drug-related liver function test (LFT) abnormalities that meets the following criteria require discontinuation:

• AST or ALT > 5 - 10×ULN for > 2 weeks

• AST or ALT > 10×ULN

• Total bilirubin > 5×ULN

• Concurrent AST or ALT > 3×ULN and total bilirubin > 2×ULN

e. Any Grade 4 drug-related adverse event or laboratory abnormality, except for the following events which do not require discontinuation:

• Grade 4 neutropenia ≤7 days

• Grade 4 lymphopenia or leukopenia

• Isolated Grade 4 electrolyte imbalances/abnormalities that are not associated with clinical sequelae and are corrected with supplementation/appropriate management within 72 hours of their onset.

• Any dosing interruption lasting > 6 weeks with the following exceptions:

√ Dosing interruptions to allow for prolonged steroid tapers to manage drug related adverse events are allowed.

√ Dosing interruptions > 6 weeks that occur for non-drug-related reasons may be allowed.

Feng K, *et al. J Immunother Cancer* 2020; 8:e000367. doi: 10.1136/jitc-2019-000367

The following events which do not require discontinuation require 20%-25% dose reduction for chemotherapeutic drugs in the next cycle treatment:

- Grade 3 or higher drug-related neutropenia
- Grade 3 or higher drug-related thrombocytopenia without association of bleeding.
- Grade 3 or higher drug-related nausea or vomiting.

5 ADVERSE EVENTS AND CLINICAL ADMINISTRATION

5.1 Definition of adverse events

An adverse event (AE) is the development of an undesirable medical condition or the deterioration of a pre-existing medical condition following or during exposure to a pharmaceutical product, whether or not be considered causally related to the product. An AE not only includes an undesirable medical condition, but also involves run-in or washout periods, even if no study treatment has been administered in clinical studies. The term of AE is used to include both serious and non-serious AEs.

5.2 Definitions of serious AE

A serious AE is an AE occurred during any study phase that meet the following criteria:

a) Results in death.

b) Is immediately life-threatening, which means the patients lies in risk of death from the AEs or it is suspected that use or continued use of the drug would result in patients' death.

Feng K, *et al. J Immunother Cancer* 2020; 8:e000367. doi: 10.1136/jitc-2019-000367

c) Requires in-patient hospitalization or prolongation of existing hospital.

d) Results in persistent or significantly disability/incapacity or disruption of the ability to conduct normal life functions.

e) Is a congenital abnormality or birth defect.

f) Is an important medical event that might need medical intervention to prevent one of the outcomes listed above.

5.3 Recording of AEs

AEs will be collected from the time of receiving the study products throughout the treatment period and the safety follow-up period. The safety follow-up period is defined as 120 days after treatment is discontinued.

After this study is finished, there might be some patients remaining on study treatment. These patients will continue to collect information about AEs.

Any AEs that are unresolved will be followed up by the investigator for as long as medically indicated. If any investigator learns of any SAEs at any time, including death, and considers there is a reasonable possibility that the events, the results need document.

5.4 Collected information for AEs

The following variables will be collected for each AE:

AEs	AE;

	The data when the AE stared and stopped;<br>Changes in CTCAE grade;<br>The serious grade of the AE;<br>Action taken with regard to investigational product;<br>Outcome;<br>Treatment measures.
SAEs	Date AE met criteria for SAE;<br>The cause of serious AE;<br>Date of hospitalization;<br>Date of discharge;<br>Causality assessment in relation to study procedures;<br>Description of AE.

All events with an assigned CTCAE grading use the grading scales found in the current National Cancer Institute CTCAE version 5.0. For those events without assigned CTCAE grades, the recommendation in the CTCAE criteria that converts mild, moderate and severe events into CTCAE grades should be used.

5.5 AEs based on signs and symptoms

All AEs that were reported by the patient or care provider in response to the open question from the study personnel, or that were revealed by observation will be collected and recorded in the CRF. When collecting AEs, the recording of diagnoses is

preferred to record a list of signs and symptoms. However, if a diagnosis is known and there are other signs or symptoms that are not generally part of the diagnosis, the diagnosis and each sign or symptom will be recorded separately.

5.6 AEs based on examination and tests

The results from protocol mandated laboratory tests and vital signs will be summarized in the clinical study report. Deterioration as compared to baseline in protocol-mandated parameters should therefore only be reported as AEs if they fulfil any of the SAE criteria or are the reason for discontinuation of treatment with the products unless due to progression of disease under study.

If deterioration in laboratory value, vital sign, ECG or other safety assessment is associated with clinical signs and symptoms, the sign or symptom will be reported as an AE and the associated laboratory result or other finding will be considered as additional information.

Deterioration of a laboratory value, which is clearly due to disease progression, should not be reported as an AE or SAE.

5.7 Disease progression

Disease progression, including the deterioration in the severity of the disease and/or increases in the symptoms of the disease, is a worsening of a patient's condition attributable to the disease for which the investigational product is being studied. The development of new, or progression of existing masses should be

considered as disease progression and not an AE. Events, which are unequivocally due to disease progression, should not be reported as an AE during the treatment period.

5.8 Report of deaths

All deaths during the study or within the follow-up period should be reported. If death is directly due to disease progression, it should be communicated to the study monitor at the next monitoring visit and should be documented in the CRF module, but should not be reported as a SAE in the study; When death is not clearly due to disease progression of the disease under study the AE causing the death should be reported to the study monitor as an SAE within 24 hours. The report should assign the primary cause of death with any contributory causes.

5.9 Management of treatment related toxicities

If subjects experience unacceptable toxicity, the subject should be withdrawn from the study and observed until resolution of the toxicity.

If subjects show a CTCAE grade≥3 toxicity during the study therapy, and the investigator consider the AE of concern to be specifically associated with investigational drugs, the treatment will be suspended, and supportive therapy administered as required in accordance with guidelines. If the toxicity resolves within 3 weeks, treatment with investigational drugs may be restarted according to the dose reduction principles.

Feng K, *et al. J Immunother Cancer* 2020; 8:e000367. doi: 10.1136/jitc-2019-000367

6 STATISTICAL ANALYSES OF TREATMENT

6.1 Statistical considerations

A comprehensive statistical analysis plan will be prepared prior to first subject enrolled. The data cut-off will be taken place at least 6 months after the last subject enrolled, to ensure that the objective of PFS at 6 months will be analyzed. Meanwhile, the primary endpoint, other secondary endpoints, and exploratory endpoints will be analyzed.

6.2 Sample size estimation

We use the A'Hern single-stage phase II study design to determine the sample size of this study. According to previously reported data, the ORR of gemcitabine and cisplatin chemotherapy for patients with advanced BTCs is approximately 26% at most. we set the null hypothesis of a proportion of patients with an objective response of 26% or lower versus the alternative hypothesis that it was 55% or higher. At least 25 patients would need to be enrolled with a two-sided significant level of 0.05 and 90% power.

6.3 Outcome measures for analysis

Response Evaluation Criteria in Solid Tumors (RECIST version 1.1)

From the investigators review of the imagining scans, RECIST version 1.1 will be used to evaluate tumor response data. It will also be used to determine the

endpoints ORR, DCR, PFS and OS from the overall visit response and scan dates.

At each evaluation, subjects will be assigned an RECIST version 1.1 response of CR, PR, SD or PD depending on the status of their disease compared with baseline assessments. If a subject has a tumor assessment which cannot be evaluated, then the subject will be assigned a visit response of not evaluable (NE) (unless there is evidence of progression in which case the response will be assigned as PD).

Overall Response Rate (ORR)：

ORR is defined as the number (%) of patients with measurable disease with at least one response evaluation of CR or PR that is confirmed at least 6 weeks later. Data obtained up until progression, or last evaluable assessment in the absence of progression, will be included in the assessment of ORR. However, any CR or PR which occurred after a further anticancer therapy will not be included in numerator of the ORR calculation.

Disease control rate (DCR)：

Disease control rate is defined as the percentage of subjects who have a CR, PR or SD.

Progression Free Survival (PFS)：

PFS is defined as the time from the first dose until the date of objective disease progression or death (by any cause in the absence of progression) regardless of whether the patient withdraws from study therapy or receives another anticancer treatment prior to progression. Subjects who have not progressed or died at the time of analysis will be censored at the time of the latest date of assessment from their last

evaluable lymphoma baseline. The PFS time will always be derived based on scan/assessment dates not visit dates.

Overall Survival (OS)：

Overall survival is defined as the time from the first dose until death due to any cause. Any patient not known to have died at the time of analysis will be censored based on the last record date on which the patients was known to be alive.

6.4 Safety Assessments

Adverse events will be listed individually by patient. Any AE occurring within 120 days of last dose of study drugs will be included in the AE summaries. For change from baseline summaries for vital signs, laboratory data and physical examination, the baseline value will be the latest result obtained prior to the start of the first dose of study drugs. All enrolled patients will be included for safety assessments.

6.5 Tumor PD-L1 assessment

Tumor samples at baseline and in the course of treatment will be obtained by excisional biopsy or with a core needle, followed by formalin fixation and paraffin embedding. tumor cell PD-L1 expression level will be assessed by immunohistochemistry using the Dako 22C3 pharmDx assay (Dako North America, Carpinteria, CA, USA). Positive tumor PD-L1 expression was defined as at least 1% of tumor cells being membrane stained at any intensity in a section that contained at

Feng K, *et al. J Immunother Cancer* 2020; 8:e000367. doi: 10.1136/jitc-2019-000367

least 100 evaluable tumor cells.

6.6 Peripheral blood T cell assessment

Blood samples will be collected for peripheral T cell assessment prior to each cycle of study treatment. The peripheral blood is collected in sodium heparin anticoagulant vacutainer tubes. Briefly, 100 µl of the anticoagulant peripheral blood is incubated with antibodies specific to cell-surface antigens expressed on T lymphocytes. After red blood cell lysis and washing, the cells are detected on a BD FACSCalibur flow cytometer (BD Biosciences). The following antibodies are used to detect surface marker expression and obtained from BD Biosciences: anti-CD3-PerCP (347344), anti-HLA-DR (559866) and isotype-matched antibodies. For the intracellular cytokine expression, blood cells are stimulated with T cell stimulation cocktail (including PMA, Ionomycin and transport inhibitors, eBioscience, 00-4975-93) for 4 h of incubation, and cells were stained with anti-CD3 and permeabilized before the addition of anti-IFN-γ (554700). Stained cells are detected by flow cytometry using FACSCalibur flow cytometer (BD Biosciences) to collect a minimum of 10,000 CD3+ lymphocytes.

6.7 Methods for statistical analyses

Appropriate descriptive statistics will be used for all variables. Continuous variables will be summarized by the number of observations, mean, standard deviation, median, minimum, and maximum. Categorical variable will be summarized

by frequency counts and percentages for each category. Unless otherwise stated, percentages will be calculated out of the full analysis set.

ORR will be presented together with two-sided 95% exact confidence interval (CI), which is calculated using the Clopper-Pearson method. Summaries of the number and percentage of patients with best response in each of the follow categories will be summarized: complete remission (CR), partial response (PR), stable disease (SD), progressive disease (PD) and non-evaluable (NE).

The best absolute change in target lesion tumor size from baseline and percentage change in target lesion tumor size from baseline will be summarized using descriptive statistics and presented at each time point.

PFS will be displayed using a Kaplan-Meier plot. The number events, median, and the proportion of patients without an event at 6 months summarized.

OS will be displayed using a Kaplan-Meier plot. The number events, median, and the proportion of patients without an event at 12, 18 months will be summarized. Summaries of the number and percentage of patients who died, still in survival follow-up, lost to follow-up or withdraw from study will be presented.

Feng K, *et al. J Immunother Cancer* 2020; 8:e000367. doi: 10.1136/jitc-2019-000367

7 LIST OF ABBREVIATIONS

AE	adverse event
ALT	alanine aminotransferase
ANC	absolute neutrophil count
AST	aspartate aminotransferase
BOR	best overall response
BTCs	biliary tract cancers
BUN	blood urea nitrogen
CI	confidence interval
cm	centimeter
CNS	central nervous system
CR	complete remission
CRF	Case Report Form
CT	Computed tomography
CTCAE	Common Terminology Criteria for Adverse Events
CTLA-4	Cytotoxic T lymphocyte-associated antigen 4
ECG	electrocardiogram
ECOG	Eastern Cooperative Oncology Group
ELISA	enzyme-linked immunosorbent assay
ESR	Expedited Safety Report
FDA	Food and Drug Administration
GCP	Good Clinical Practice

Feng K, *et al. J Immunother Cancer* 2020; 8:e000367. doi: 10.1136/jitc-2019-000367

HBV	hepatitis B virus
HCV	hepatitis C virus
HRP	horseradish peroxidase
ICF	Informed Consent
ICH	International Conference on Harmonisation
IHC	immunohistochemistry
IRB	Institutional Review Board
IV	Intravenous
Kg	kilogram
MRI	Magnetic Resonance Imaging
mAbs	Monoclonal Antibodys
mg/kg	Milligram per kilogram
NE	non-evaluable
ORR	Objective Response Rate
OS	Overall survival
PBMCs	peripheral blood mononuclear cells
PD	progressive disease
PD-1	Programmed death 1
PD-L1	Programmed death ligand 1
PD-L2	Programmed death ligand 2
PET	Positron emission tomography

Feng K, *et al. J Immunother Cancer* 2020; 8:e000367. doi: 10.1136/jitc-2019-000367

PFS	progression free survival
PR	partial remission
PS	Performance Status
RECIST	Response Evaluation Criteria in Solid Tumors
RT-PCR	reverse transcription polymerase chain reaction
RNA	Ribonucleic acid
SAE	serious adverse event
SD	Stable Disease
TCR	T cell receptor
WBC	white blood cell
WHO	World Health Organization

8 REFERENCE

1. Bray F, Ferlay J, Soerjomataram I, Siegel RL, Torre LA, Jemal A. Global cancer statistics 2018: GLOBOCAN estimates of incidence and mortality worldwide for 36 cancers in 185 countries. CA Cancer J Clin. 2018;68(6):394-424.

2. GBD 2016 Disease and Injury Incidence and Prevalence Collaborators. Global, regional, and national incidence, prevalence, and years lived with disability for 328 diseases and injuries for 195 countries, 1990-2016: a systematic analysis for the Global Burden of Disease Study 2016. Lancet. 2017; 390(10100):1211-1259.

3. Razumilava N, Gores GJ. Classification, Diagnosis, and Management of Cholangiocarcinoma. Clin Gastroenterol Hepatol. 2013; 11(1): 13–e4.

4. Valle J, Wasan H, Palmer DH, Cunningham D, Anthoney A, Maraveyas A, et al. Cisplatin plus Gemcitabine versus Gemcitabine for Biliary Tract Cancer. N Engl J Med. 2010;362(14):1273-1281.

5. Malka D, Cervera P, Foulon S, Trarbach T, de la Fouchardière C, Boucher E, et al. Gemcitabine and oxaliplatin with or without cetuximab in advanced biliary-tract cancer (BINGO): a randomised, open-label, non-comparative phase 2 trial. Lancet Oncol. 2014;15(8):819-828.

6. Park K, Kim KP, Park S, Chang HM. Comparison of gemcitabine plus cisplatin versus capecitabine plus cisplatin as first-line chemotherapy for advanced biliary tract cancer. Asia Pac J Clin Oncol. 2017;13(1):13-20.

7. Rizvi S, Gores GJ. Emerging molecular therapeutic targets for cholangiocarcinoma. J Hepatol. 2017;67(3):632-644.

8. Heymach J, Krilov L, Alberg A, Baxter N, Chang SM, Corcoran R, et al. Clinical Cancer Advances 2018: Annual Report on Progress Against Cancer from the American Society of Clinical Oncology. J Clin Oncol. 2018;36(10):1020-1044.

9. Martin-Liberal J, Ochoa de Olza M, Hierro C, Gros A, Rodon J, Tabernero J. The expanding role of immunotherapy. Cancer Treat Rev. 2017; 54:74-86.

10. Gani F, Nagarajan N, Kim Y, Zhu Q, Luan L, Bhaijjee F, et al. Program Death 1 Immune Checkpoint and Tumor Microenvironment: Implications for Patients With Intrahepatic Cholangiocarcinoma. Ann Surg Oncol. 2016;23(8):2610-2617.

11. Sabbatino F, Villani V, Yearley JH, Deshpande V, Cai L, Konstantinidis IT, et al. PD-L1 and HLA Class I Antigen Expression and Clinical Course of the Disease in Intrahepatic Cholangiocarcinoma. Clin Cancer Res. 2016;22(2):470-478.

12. Nakamura H, Arai Y, Totoki Y, Shirota T, Elzawahry A, Kato M, et al. Genomic spectra of biliary tract cancer. Nat Genet. 2015;47(9):1003-1010.

13.Bang YJ, Doi T, De Braud F, et al. Safety and efficacy of pembrolizumab (MK-3475) in patients (pts) with advanced biliary tract cancer: interim results of KEYNOTE-028. Eur J Cancer 2015;51(3): s112.

14. Le DT, Durham JN, Smith KN, Wang H, Bartlett BR, Aulakh LK, et al. Mismatch repair deficiency predicts response of solid tumors to PD-1 blockade. Science. 2017;357(6349):409-413.

15. Silva VW, Askan G, Daniel TD, Lowery M, Klimstra DS, Abou-Alfa GK, et al. Biliary carcinomas: pathology and the role of DNA mismatch repair deficiency. Chin Clin Oncol. 2016;5(5):62.

16. Wumkes ML, van der Velden AM, Los M, Leys MB, Beeker A, Nijziel MR, et al. Serum antibody response to influenza virus vaccination during chemotherapy treatment in adult patients with solid tumors. Vaccine. 2013;31(52):6177-6184.

17. Garg AD, Vara Perez M, Schaaf M, Agostinis P, Zitvogel L, Kroemer G, et al.Trial watch: Dendritic cell-based anticancer immunotherapy. Oncoimmunology. 2017;6(7): e1328341.

18. Galluzzi L, Buqué A, Kepp O, Zitvogel L, Kroemer G. Immunological Effects of Conventional Chemotherapy and Targeted Anticancer Agents. Cancer Cell. 2015;28(6):690-714.

19. Brahmer JR, Drake CG, Wollner I, Powderly JD, Picus J, Sharfman WH, et al. Phase I study of single-agent anti-programmed death-1 (MDX-1106) in refractory solid tumors: safety, clinical activity, pharmacodynamics, and immunologic correlates. J Clin Oncol. 2010;28(19):3167-3175.

20. Di Caro G, Cortese N, Castino GF, Grizzi F, Gavazzi F, Ridolfi C, et al. Dual prognostic significance of tumor-associated macrophages in human pancreatic adenocarcinoma treated or untreated with chemotherapy. Gut. 2016;65(10):1710-1720.

21. Liu WM, Fowler DW, Smith P, Dalgleish AG. Pre-treatment with chemotherapy can enhance the antigenicity and immunogenicity of tumors by promoting adaptive immune responses. Br J Cancer. 2010;102(1):115-123.

22. Lesterhuis WJ, Punt CJ, Hato SV, Eleveld-Trancikova D, Jansen BJ, Nierkens S, et al. Platinum-based drugs disrupt STAT6-mediated suppression of immune

responses against cancer in humans and mice. J Clin Invest. 2011;121(8):3100-3108.

23. Chen C, Chen Z, Chen D, Zhang B, Wang Z, Le H. Suppressive effects of gemcitabine plus cisplatin chemotherapy on regulatory T cells in non-small cell lung cancer. J Int Med Res. 2015;43(2):180-187.

APPENDIX 1 Response Evaluation Criteria in Solid Tumors version 1.1

1.1 Time point response: patients with target (+/–non-target) disease

Target lesions	Non-target lesions	New lesions	Overall response
CR	CR	No	CR
CR	Non-CR/non-PD	No	PR
CR	Not evaluated	No	PR
PR	Non-PD or not all evaluated	No	PR
SD	Non-PD or not all evaluated	No	SD
Not all evaluated	Non-PD	No	NE
PD	Any	Yes or No	PD
Any	PD	Yes or No	PD
Any	Any	Yes	PD
CR = complete response, PR = partial response, SD = stable disease, PD = progressive disease, and NE = inevaluable.			

1.2 Response criteria

1.2.1 Evaluation of target lesions

Complete Response (CR): Disappearance of all target lesions. Any pathological lymph nodes (whether target or non-target) must have reduction in short axis to <10 mm.

Partial Response (PR): At least a 30% decrease in the sum of diameters of target lesions, taking as reference the baseline sum diameters.

Progressive Disease (PD): At least a 20% increase in the sum of diameters of target lesions, taking as reference the smallest sum on study (this includes the baseline sum if that is the smallest on study). In addition to the relative increase of 20%, the

Feng K, *et al. J Immunother Cancer* 2020; 8:e000367. doi: 10.1136/jitc-2019-000367

sum must also demonstrate an absolute increase of at least 5 mm. (Note: the appearance of one or more new lesions is also considered progression).

Stable Disease (SD): Neither sufficient shrinkage to qualify for PR nor sufficient increase to qualify for PD, taking as reference the smallest sum diameters while on study.

1.2.2 Evaluation of non-target lesions

Complete Response (CR): Disappearance of all non-target lesions and normalization of tumor marker level. All lymph nodes must be non-pathological in size (<10mm short axis).

Non-CR/Non-PD: Persistence of one or more non-target lesion (s) and/or maintenance of tumor marker level above the normal limits.

Progressive Disease (PD): Unequivocal progression (see comments below) of existing non-target lesions. (Note: the appearance of one or more new lesions is also considered progression).

Feng K, *et al. J Immunother Cancer* 2020; 8:e000367. doi: 10.1136/jitc-2019-000367

APPENDIX 2 COMMON TERMINOLOGY CRITERIA FOR ADVERSE EVENTS V5.0 (CTCAE)

The descriptions and grading scales found in the revised NCI Common Terminology Criteria for Adverse Events (CTCAE) version 5.0 will be utilized for adverse event reporting(http://ctep.cancer.gov/protocolDevelopment/electronic_applications/ctc.htm).

Feng K, *et al. J Immunother Cancer* 2020; 8:e000367. doi: 10.1136/jitc-2019-000367

APPENDIX 3 ECOG PERFORMANCE STATUS SCALE

Grade	Description
0	Normal activity. Fully active, able to carry on all pre-disease performance without restriction.
1	Symptoms, but ambulatory. Restricted in physically strenuous activity, but ambulatory and able to carry out work of a light or sedentary nature (e.g., light housework, office work).
2	In bed <50% of the time. Ambulatory and capable of all self-care, but unable to carry out any work activities. Up and about more than 50% of waking hours.
3	In bed >50% of the time. Capable of only limited self-care, confined to bed or chair more than 50% of waking hours.
4	100% bedridden. Completely disabled. Cannot carry on any self-care. Totally confined to bed or chair.
5	Dead.

Feng K, *et al. J Immunother Cancer* 2020; 8:e000367. doi: 10.1136/jitc-2019-000367

APPENDIX 4 NEW YORK HEART ASSOCIATION (NYHA) CLASSIFICATION

Class I: patients with no limitation of activities; they suffer no symptoms from ordinary activities.

Class II: patients with slight, mild limitation of activity; they are comfortable with rest or with mild exertion.

Class III: patients with marked limitation of activity; they are comfortable only at rest.

Class IV: patients who should be at complete rest, confined to bed or chair; any physical activity brings on discomfort and symptoms occur at rest.

Feng K, *et al. J Immunother Cancer* 2020; 8:e000367. doi: 10.1136/jitc-2019-000367

SUMMARY OF PROTOCOL CHANGES

**Document**	**Date of Issue**	**Summary of changes and amendments**
Final Version (version 2)	May 15, 2017	Eliminate maintenance therapy with nivolumab alone; Add a second objective OS at 12 months; Assess tumor PD-L1 expression with immunohistochemistry using the Dako 22C3 pharmDx assay; Clarify the statistical determination of sample size; Check the spelling, grammar errors.
Original Version (version 1)	April 1, 2017	Not applicable

Feng K, *et al. J Immunother Cancer* 2020; 8:e000367. doi: 10.1136/jitc-2019-000367
