# Supplementary material for: Efficacy and biomarker analysis of nivolumab plus gemcitabine and cisplatin in patients with unresectable or metastatic biliary tract cancers: results from a phase II study
Source: J Immunother Cancer. 2020 Jun 2;8(1):e000367. doi: 10.1136/jitc-2019-000367 (PMC7269541; doi:10.1136/jitc-2019-000367)
Supplement: Supplementary data [file jitc-2019-000367supp002.pdf]

## Supplementary Data:

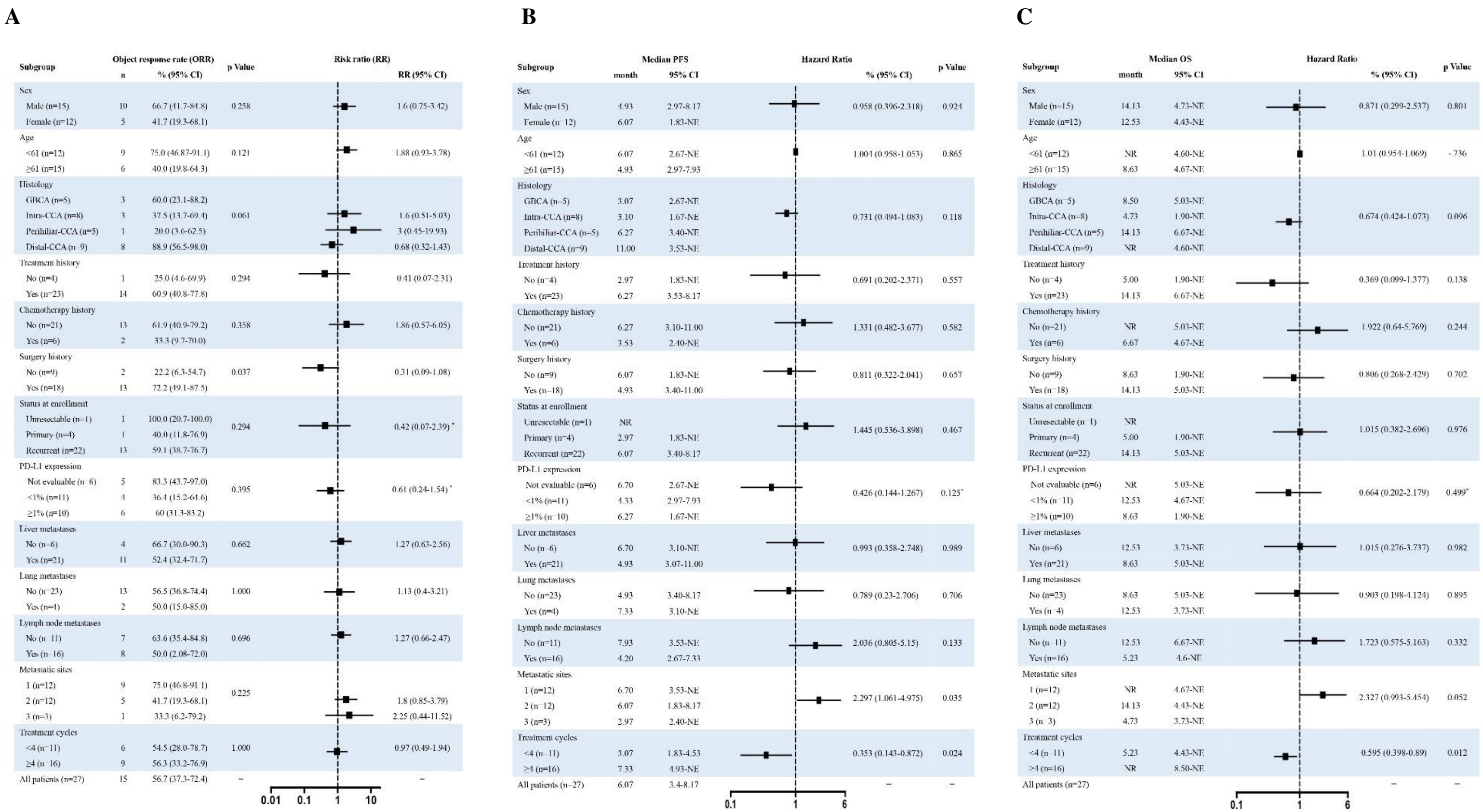


**Figure S1:** Multivariate analysis of the correlation between different variables and ORR (A), median PFS (B) and median OS (C).



Feng K, *et al. J Immunother Cancer* 2020; 8:e000367. doi: 10.1136/jitc-2019-000367

| Sample | MSI | TMB | TNB | Fitness | Heterogeneity | Group |
|---|---|---|---|---|---|---|
| Pt19 | 2.13 | 2.85 | 1.39 | -15.789 | NA | Respond |
| Pt30 | 1.82 | 2.96 | 1.12 | -16.6964 | 0.2 | Respond |
| Pt13 | 0.1 | 1.33 | 0.65 | -4.37534 | 0.84 | NonRespond |
| Pt4 | 2.67 | 4.65 | 2.03 | -8.96791 | 0 | Respond |
| Pt17 | 0.74 | 4.5 | 1.5 | -14.869 | 0.16 | NonRespond |
| Pt27 | 2.76 | 6.83 | 4.07 | -7.33157 | 0.13 | NA |
| Pt25 | 1.61 | 2.56 | 1.37 | -2.16677 | 0.32 | NonRespond |
| Pt8 | 0.7 | 3.24 | 1.36 | -3.42382 | NA | NonRespond |
| Pt24 | 0.71 | 0.94 | 0.25 | -1.7125 | NA | NonRespond |
| Pt10 | 3.12 | 0.87 | 0.52 | -0.47016 | 0.28 | NA |
| Pt6 | 0.71 | 1.91 | 0.5 | -2.01753 | 0.47 | NonRespond |
| Pt16 | 2.11 | 1.87 | 0.87 | -11.7561 | 0.98 | NA |
| Pt20 | 2.07 | 1.46 | 0.94 | -6.9884 | 0.82 | NonRespond |
| Pt31 | 1.19 | 3.37 | 1.63 | -7.1236 | NA | Respond |
| Pt28 | 1.43 | 1.63 | 0.53 | -14.8748 | 0.25 | Respond |
| Pt14 | 2.11 | 6.28 | 2.24 | -1.47346 | 0.97 | Respond |
| Pt7 | 2.82 | 4.46 | 2.26 | -1.97912 | 0.3 | NonRespond |
| Pt23 | 1.39 | 1.39 | 0.97 | -13.5947 | NA | Respond |
| Pt18 | 1.29 | 1.3 | 0.68 | -5.94804 | 0.4 | NonRespond |
| Pt2 | 2.1 | 2.94 | 1.23 | -2.39602 | 0.12 | Respond |

**Figure S2:** Data of whole-exome sequencing.



Feng K, *et al. J Immunother Cancer* 2020; 8:e000367. doi: 10.1136/jitc-2019-000367

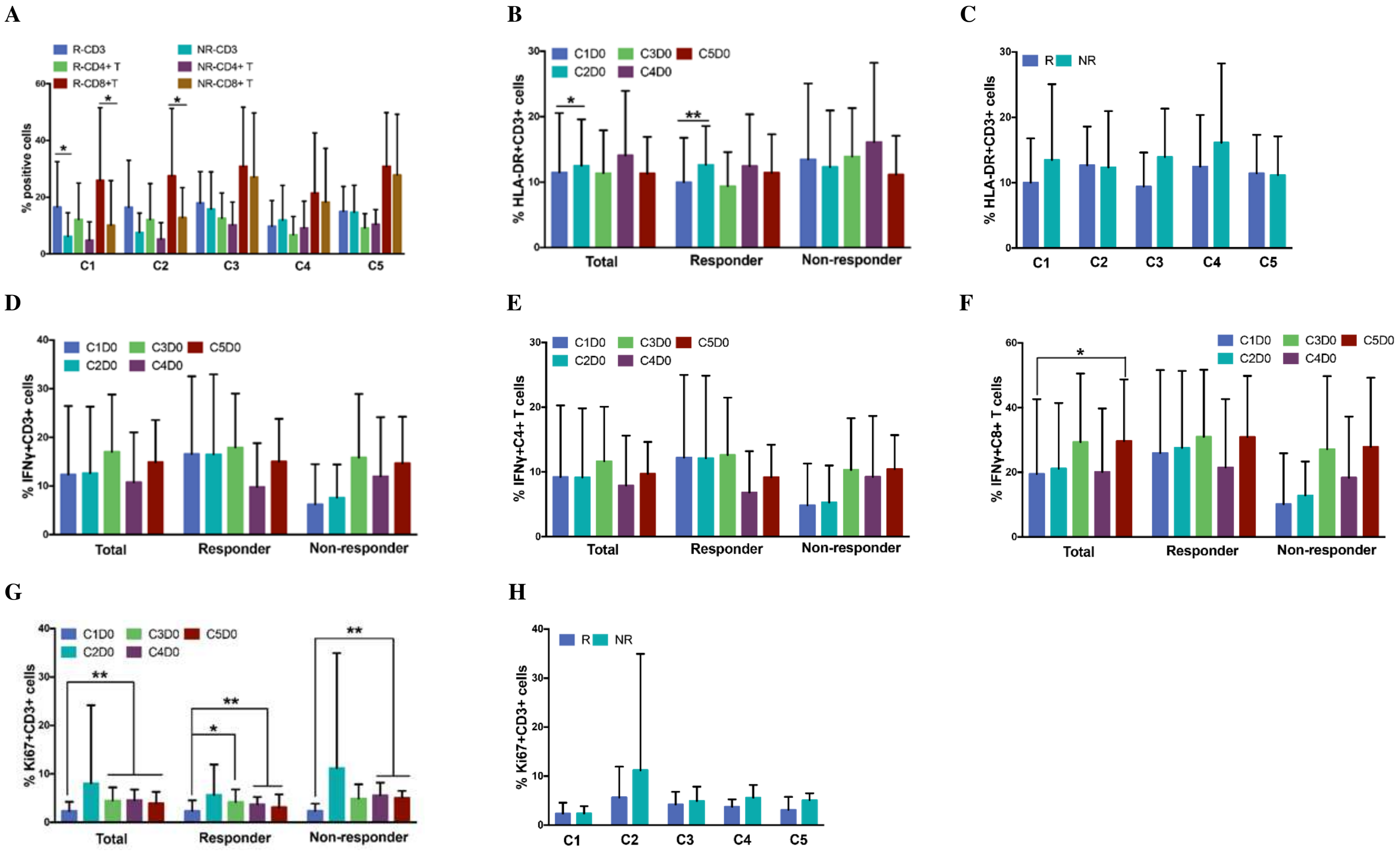


**Figure S3:** Analysis of the association between clinical antitumor activity and the activation of peripheral T cells.

* : P ≤ 0.05.  * * : P ≤ 0.01. R = responder. NR = non-responder.



Feng K, *et al. J Immunother Cancer* 2020; 8:e000367. doi: 10.1136/jitc-2019-000367

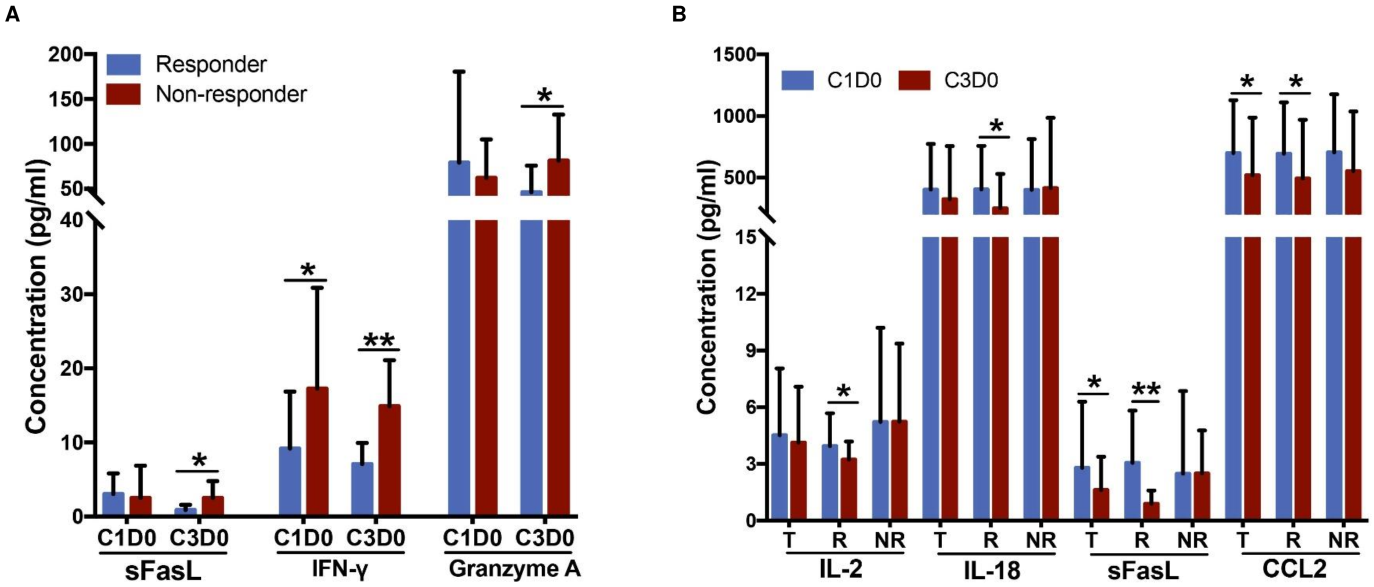


**Figure S4:** Analysis of the association between clinical antitumor activity and the concentration of serum cytokines and chemokines

* : P ≤ 0.05. * * : P ≤ 0.01. T = total. R = responder. NR = non-responder.

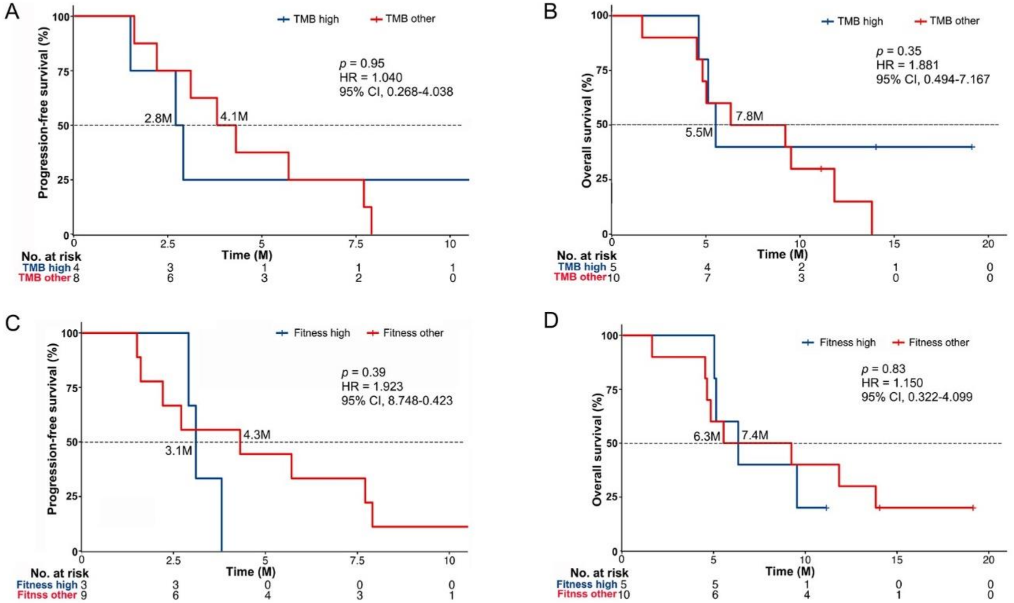


**Figure S5:** A. Kaplan-Meier curves of progression free survival of patients with high TMB and other levels of TMB. B. Kaplan-Meier curves of overall survival of patients with high TMB and other levels of TMB. C. Kaplan-Meier curves of progression free survival of patients with high fitness and other levels of fitness. D. Kaplan-Meier curves of overall survival of patients with high fitness and other levels of fitness.



Feng K, *et al. J Immunother Cancer* 2020; 8:e000367. doi: 10.1136/jitc-2019-000367

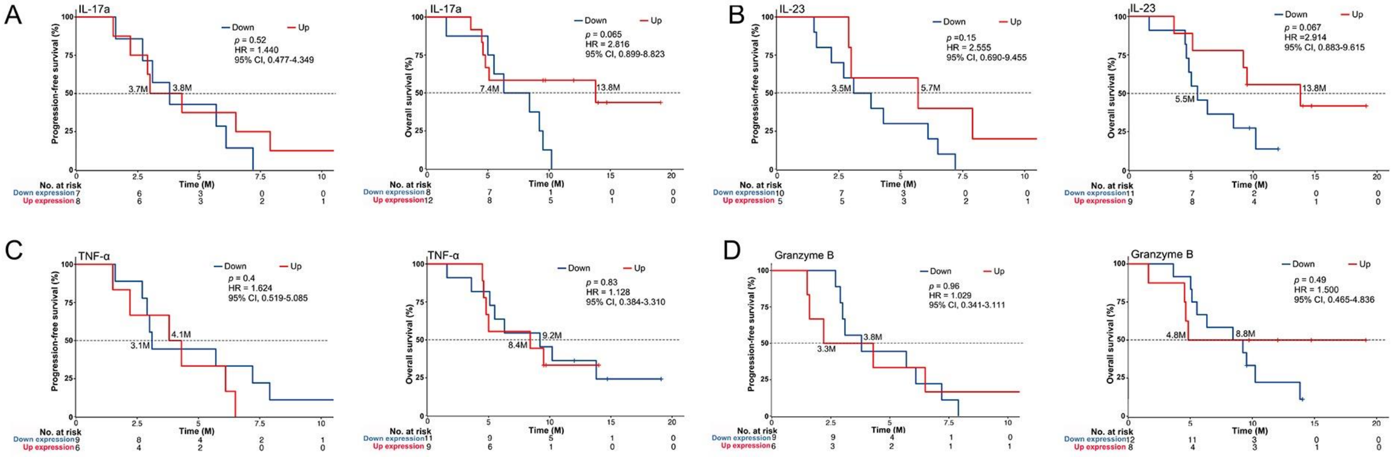


**Figure S6:** Analysis of correlation between serum IL-17a (A), IL-23 (B), TNF-α (C) and Granzyme B (D)and progression free survival and overall survival.

**Figure S7**

**Patient 1 with SD**

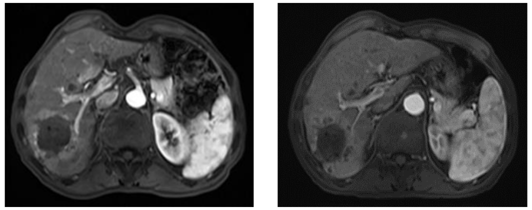

Baseline    4-cycle after treatment

**Patient 2 with PR**

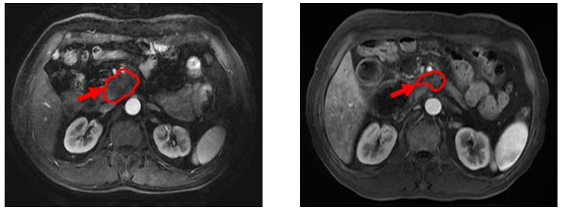

Baseline    2-cycle after treatment

**Patient 4 with CR**

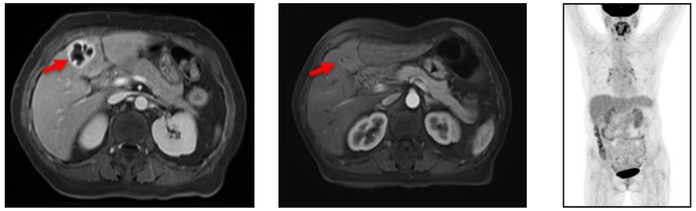

Baseline    4-cycle after treatment    PET/CT confirmation

**Patient 5 with PR**

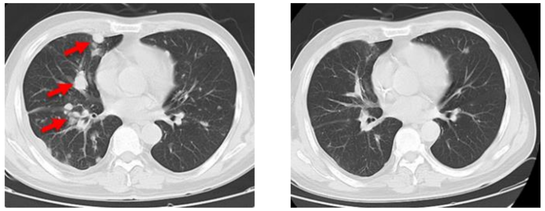

Baseline    2-cycle after treatment



Feng K, *et al. J Immunother Cancer* 2020; 8:e000367. doi: 10.1136/jitc-2019-000367

**Patient 6 with SD**

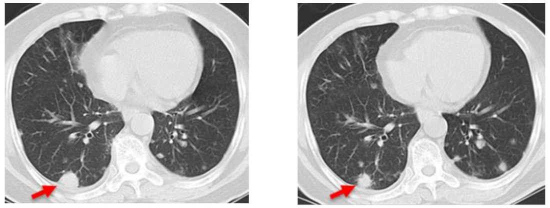

Baseline 6-cycle after treatment

**Patient 7 with SD**

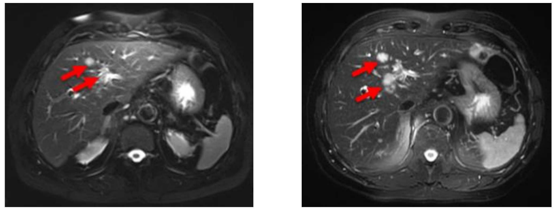

Baseline 4-cycle after treatment

**Patient 8 with PD**

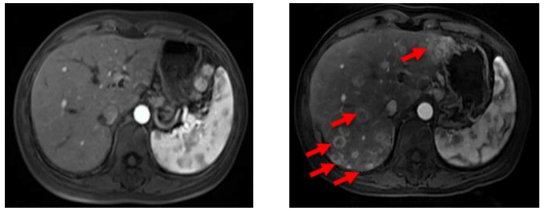

Baseline 2-cycle after treatment

**Patient 9 with PR**

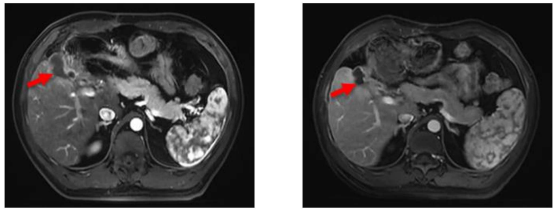

Baseline 2-cycle after treatment

**Patient 11 with SD**

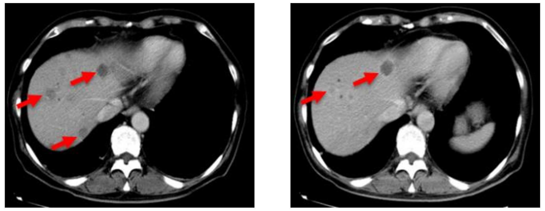

**Patient 12 with CR**

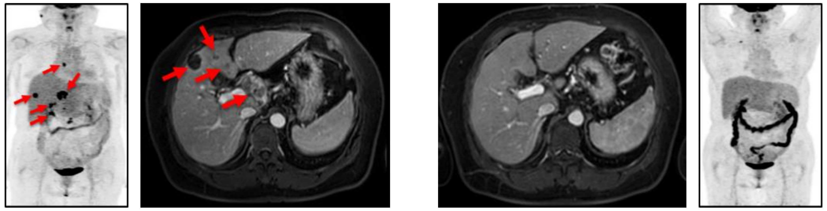



Feng K, *et al. J Immunother Cancer* 2020; 8:e000367. doi: 10.1136/jitc-2019-000367

Baseline 2-cycle after treatment

**Patient 13 with SD**

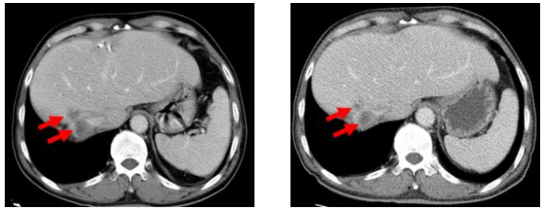

Baseline PET/CT Baseline 6-cycle after treatment PET/CT confirmation

**Patient 14 with CR**

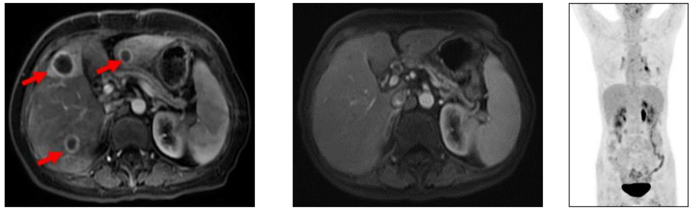

Baseline 4-cycle after treatment

**Patient 15 with PR**

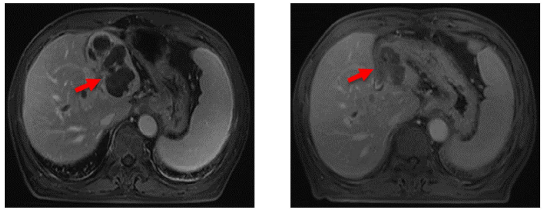

Baseline 6-cycle after treatment PET/CT confirmation

**Patient 17 with SD**

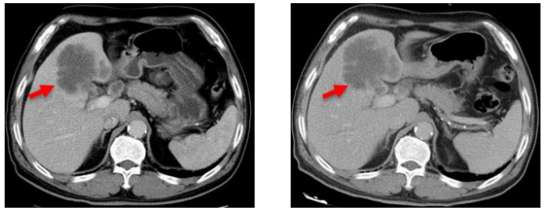

Baseline 6-cycle after treatment

**Patient 18 with SD**

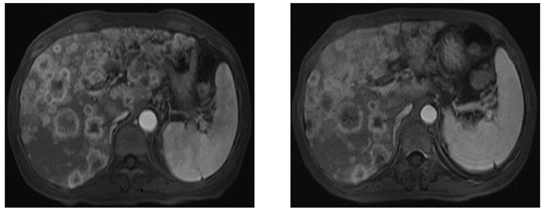

Baseline 2-cycle after treatment

**Patient 19 with CR**

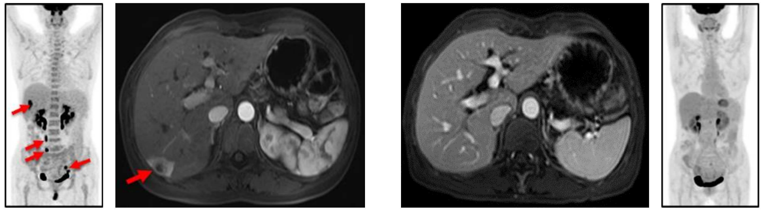



Feng K, *et al. J Immunother Cancer* 2020; 8:e000367. doi: 10.1136/jitc-2019-000367

Baseline 2-cycle after treatment Baseline PET/CT Baseline 6-cycle after treatment PET/CT confirmation

**Patient 20 with PD**

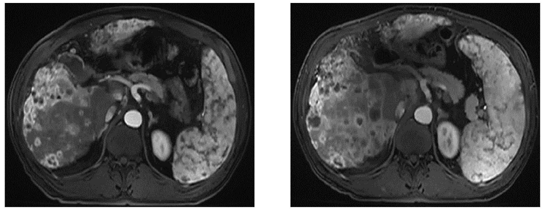

Baseline 2-cycle after treatment

**Patient 21 with SD**

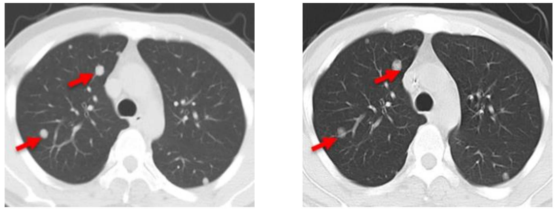

Baseline 6-cycle after treatment

**Patient 22 with CR**

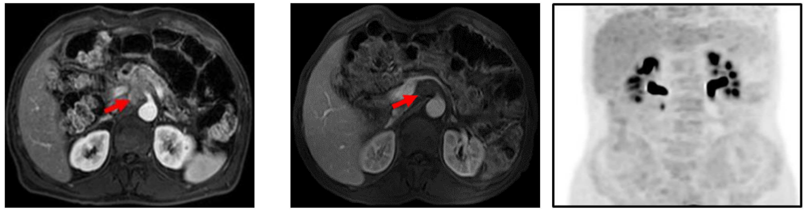

Baseline 6-cycle after treatment PET/CT confirmation

**Patient 23 with PR**

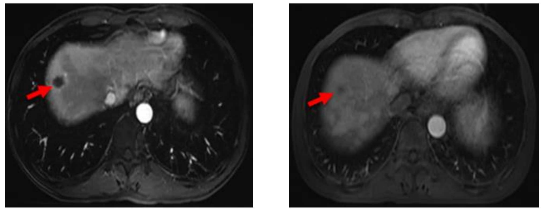

Baseline 2-cycle after treatment

**Patient 24 with SD**

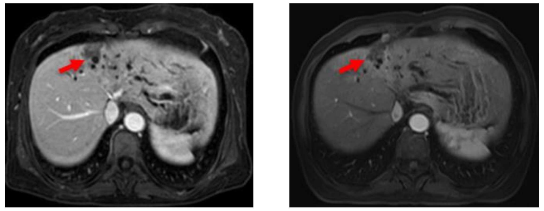

**Patient 25 with SD**

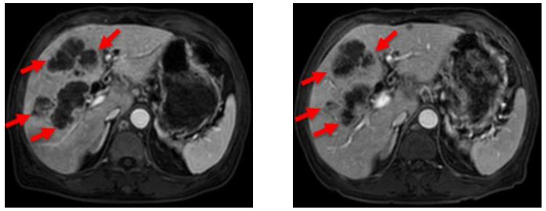

Baseline 2-cycle after treatment

Baseline 2-cycle after treatment

**Patient 26 with PR**

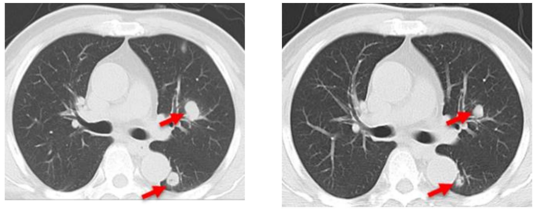

Baseline 2-cycle after treatment

**Patient 28 with PR**

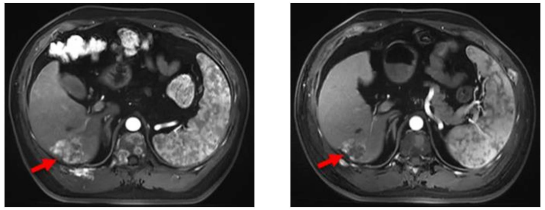

Baseline 2-cycle after treatment

**Patient 30 with PR**

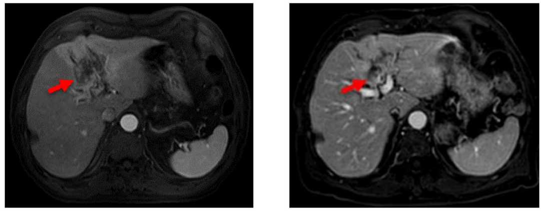

Baseline 2-cycle after treatment

**Patient 31 with PR**

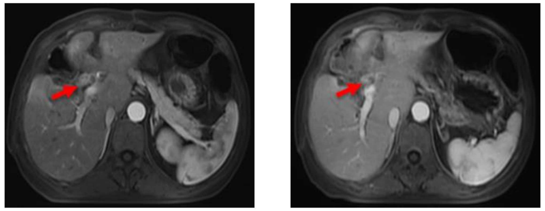

Baseline 2-cycle after treatment

**Patient 32 with PR**

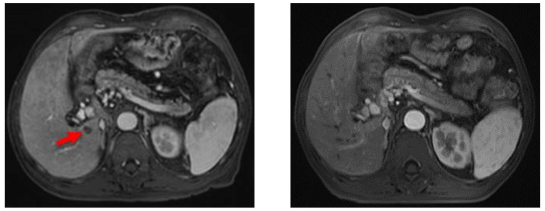

Baseline 2-cycle after treatment



Feng K, *et al. J Immunother Cancer* 2020; 8:e000367. doi: 10.1136/jitc-2019-000367
